# Enhanced oxidative stress in smoking and ex-smoking severe asthma in the U-BIOPRED cohort

**DOI:** 10.1371/journal.pone.0203874

**Published:** 2018-09-21

**Authors:** Rosalia Emma, Aruna T. Bansal, Johan Kolmert, Craig E. Wheelock, Swen-Erik Dahlen, Matthew J. Loza, Bertrand De Meulder, Diane Lefaudeux, Charles Auffray, Barbro Dahlen, Per S. Bakke, Pascal Chanez, Stephen J. Fowler, Ildiko Horvath, Paolo Montuschi, Norbert Krug, Marek Sanak, Thomas Sandstrom, Dominick E. Shaw, Louise J. Fleming, Ratko Djukanovic, Peter H. Howarth, Florian Singer, Ana R. Sousa, Peter J. Sterk, Julie Corfield, Ioannis Pandis, Kian F. Chung, Ian M. Adcock, René Lutter, Lorena Fabbella, Massimo Caruso, U-BIOPRED Study Group

**Affiliations:** 1 Department of Clinical and Experimental Medicine – University of Catania, Catania, Italy; 2 Acclarogen Ltd, St John’s Innovation Centre, Cambridge, United Kingdom; 3 Division of Physiological Chemistry 2, Department of Medical Biochemistry and Biophysics, Karolinska Institutet, Stockholm, Sweden; 4 Centre for Allergy Research, Institute of Environmental Medicine, Karolinska Institutet, Stockholm, Sweden; 5 Janssen Research & Development, LLC, Springhouse, Pennsylvania, United States of America; 6 European Institute for Systems Biology and Medicine, CNRS-ENS-UCBL-INSERM, CIRI-UMR5308, Lyon, France; 7 Karolinska University Hospital & Centre for Allergy Research, Karolinska Institutet, Stockholm, Sweden; 8 Department of Clinical Science, University of Bergen, Bergen, Norway; 9 Département des Maladies Respiratoires, CIC Nord, INSERM U1067 Aix Marseille Université Marseille, Marseille, France; 10 Centre for Respiratory Medicine and Allergy, The University of Manchester, Manchester Academic Health Science Centre, University Hospital of South Manchester NHS Foundation Trust, Clinic, Lancashire Teaching Hospitals NHS Foundation Trust, Preston, United Kingdom; 11 Department of Pulmonology, Semmelweis University, Budapest, Hungary; 12 Faculty of Medicine, Catholic University of the Sacred Heart, Rome, Italy; 13 Fraunhofer Institute for Toxicology and Experimental Medicine Hannover, Germany; 14 Department of Medicine, Jagiellonian University Medical School, Krakow, Poland; 15 Dept of Public Health and Clinical Medicine, Medicine, Umeå University, Umeå, Sweden; 16 Respiratory Research Unit, University of Nottingham, Nottingham, United Kingdom; 17 National Heart & Lung Institute, Imperial College, London, United Kingdom; 18 NIHR Southampton Respiratory Biomedical Research Unit, Clinical and Experimental Sciences, University of Southampton Faculty of Medicine, Southampton, United Kingdom; 19 University Children’s Hospital Bern, Bern, Switzerland; 20 University Children’s Hospital Zurich, Zurich, Switzerland; 21 Respiratory Therapy Unit, GlaxoSmithKline, London, United Kingdom; 22 Dept of Respiratory Medicine, Academic Medical Centre, University of Amsterdam, Amsterdam, The Netherlands; 23 AstraZeneca R&D, Mölndal, Sweden; 24 Areteva R&D, Nottingham, United Kingdom; 25 Data Science Institute, South Kensington Campus, Imperial College London, London, United Kingdom; 26 Department of Biomedical and Biotechnological Sciences (BIOMETEC), University of Catania, Catania, Italy; National and Kapodistrian University of Athens, GREECE

## Abstract

Oxidative stress is believed to be a major driver of inflammation in smoking asthmatics. The U-BIOPRED project recruited a cohort of Severe Asthma smokers/ex-smokers (SAs/ex) and non-smokers (SAn) with extensive clinical and biomarker information enabling characterization of these subjects. We investigated oxidative stress in severe asthma subjects by analysing urinary 8-iso-PGF_2α_ and the mRNA-expression of the main pro-oxidant (NOX2; NOSs) and anti-oxidant (SODs; CAT; GPX1) enzymes in the airways of SAs/ex and SAn. All the severe asthma U-BIOPRED subjects were further divided into current smokers with severe asthma (CSA), ex-smokers with severe asthma (ESA) and non-smokers with severe asthma (NSA) to deepen the effect of active smoking. Clinical data, urine and sputum were obtained from severe asthma subjects. A bronchoscopy to obtain bronchial biopsy and brushing was performed in a subset of subjects. The main clinical data were analysed for each subset of subjects (urine-8-iso-PGF_2α_; IS-transcriptomics; BB-transcriptomics; BBr-transcriptomics). Urinary 8-iso-PGF_2α_ was quantified using mass spectrometry. Sputum, bronchial biopsy and bronchial brushing were processed for mRNA expression microarray analysis. Urinary 8-iso-PGF_2α_ was increased in SAs/ex, median (IQR) = 31.7 (24.5–44.7) ng/mmol creatinine, compared to SAn, median (IQR) = 26.6 (19.6–36.6) ng/mmol creatinine (*p*< 0.001), and in CSA, median (IQR) = 34.25 (24.4–47.7), *vs*. ESA, median (IQR) = 29.4 (22.3–40.5), and NSA, median (IQR) = 26.5 (19.6–16.6) ng/mmol creatinine (*p* = 0.004). Sputum mRNA expression of NOX2 was increased in SAs/ex compared to SAn (probe sets 203922_PM_s_at fold-change = 1.05 *p* = 0.006; 203923_PM_s_at fold-change = 1.06, *p* = 0.003; 233538_PM_s_at fold-change = 1.06, *p* = 0.014). The mRNA expression of antioxidant enzymes were similar between the two severe asthma cohorts in all airway samples. NOS2 mRNA expression was decreased in bronchial brushing of SAs/ex compared to SAn (fold-change = -1.10; *p* = 0.029). NOS2 mRNA expression in bronchial brushing correlated with FeNO (Kendal’s Tau = 0.535; *p*< 0.001). From clinical and inflammatory analysis, FeNO was lower in CSA than in ESA in all the analysed subject subsets (*p*< 0.01) indicating an effect of active smoking. Results about FeNO suggest its clinical limitation, as inflammation biomarker, in severe asthma active smokers. These data provide evidence of greater systemic oxidative stress in severe asthma smokers as reflected by a significant changes of NOX2 mRNA expression in the airways, together with elevated urinary 8-iso-PGF_2α_ in the smokers/ex-smokers group.

**Trial registration** ClinicalTrials.gov—Identifier: NCT01976767

## Introduction

Asthma is a heterogeneous inflammatory syndrome of the airways characterized by several clinical and molecular phenotypes [1–3]. In severe asthma (SA), genetic, immunologic and environmental factors interact contributing to airway chronic inflammation [4]. Cigarette smoke is a key factor implicated in modulation of asthma. Data on asthmatic smokers suggest marked impairment in asthma control, accelerated decline in lung function, increased airflow obstruction and increase in disease severity [5].

Human airways are normally exposed to oxidative products present in environmental pollutants. But, the inflammatory state in the airways of asthmatic patients may also promote oxidative stress with increased levels of reactive oxygen species (ROS) and reactive nitrogen species (RNS)[6], which may further contribute to maintenance and progression of the inflammatory response and disease exacerbation [7]. Activated inflammatory cells produce anion superoxide (O2•^-^) through the NADPH oxidase pathway. The O2•^-^ is neutralized by superoxide dismutase enzymes (SODs), catalase (CAT) and glutathione peroxidase (GPX) activity [8]. Furthermore, nitric oxide synthase (NOS) enzymes generate nitric oxide (NO)[9], another common free radical, that in the presence of ROS rapidly forms RNS [8]. An excess of ROS and RNS has been shown to lead to membrane lipids peroxidation, nicotinamide nucleotides depletion, enhanced intracellular Ca^2+^, cytoskeleton breakage and DNA damage [8,10]. The peroxidative breakdown of membrane fatty acids by ROS leads to F_2_-Isoprostanes production. 8-Isoprostaglandin F2α (8-iso-PGF_2α_) is therefore considered a useful marker of oxidative stress [11].

Tobacco smoke is a major exogenous source of oxidative stress, contributing to subsistence and progression of the inflammatory response and disease chronicity in asthma. Many oxidant compounds are present in cigarette smoke, which may induce direct and/or indirect oxidative damage [12]. Very little is known about the role of oxidative stress in SA and even less about the combined impact with cigarette smoking. We tested the hypothesis that oxidative stress and inflammatory biomarkes differs in SA subjects with and without a significant smoking history, as well as in SA current-, ex- and non-smokers.

We therefore investigated the level of the lipid peroxidation marker 8-iso-PGF_2α_ in the urine and the mRNA expression profile of key pro-oxidant (NADPH oxidase 2, NOX2; inducible NOS, NOS2; constitutive NOSs, NOS1 and NOS3) and anti-oxidant (superoxide dismutases, SOD1, -2 and -3; catalase, CAT; and glutathione peroxidase 1, GPX1) enzymes in the airways, in particular in bronchial biopsy (BB), bronchial brushing (BBr) and induced sputum (IS) samples.

## Materials and methods

Materials and methods section is fully described in the online S1 File. Material and Methods.

### Subjects

The Severe Asthma U-BIOPRED participants were enrolled in two groups [13]:

- *Severe non-smoking asthma (SAn)*: subjects in this group refrained from smoking for at least 12 months prior to the study, with a less than five pack-years smoking history. They had uncontrolled symptoms as defined according to GINA guidelines [14] and/or frequent exacerbations (more than two per year) despite high-dose inhaled corticosteroids (ICS ≥ 1000 μg fluticasone propionate/day or equivalent dose).
- *Smokers and ex-smokers with severe asthma (SAs/ex)*: this group was defined as for the SAn group except that they were either current smokers or ex-smokers with at least five pack-years smoking history.

In order to deepen potential effect of current smoking, we further divided all the severe asthma U-BIOPRED subjects into three subgroups by smoking status: Current smokers with Severe Asthma (CSA); Ex-smokers with Severe Asthma (ESA); Non-smokers with Severe Asthma (NSA).

All enrolled subjects underwent a baseline visit during which clinical data, and urine and sputum samples were collected. An optional broncoscopy visit was carried out only in specialist centres. Clinical and omic data of severe asthma cohorts were downloaded on June 2016 from U-BIOPRED database (tranSMART system) [15]. We obtained urine 8-iso-PGF_2α_ data from 411 severe asthma subjects, and IS, BB, BBr transcriptomincs data from 84, 53, 67 severe asthma subjects, respectively. There is a good overlap, in term of patient coverage between BB, BBr and urine, and between sputum and urine. In contrast, there is little overlap between patients who had IS and BB/BBr.

The study protocol was approved by the Ethics Review Board of the Academic Medical Centre of the University of Amsterdam (The Netherlands) and subsequently by the Ethics Boards of all other clinical centres in the study (see S1 File. Material and Methods). The study adhered to the standards set by International Conference on Harmonisation and Good Clinical Practice. All participants signed a written informed consent.

### 8-iso-PGF_2α_ assessment

8-iso-PGF_2α_ was extracted from spot urine samples using solid phase extraction (SPE) and quantified via liquid chromatography coupled to mass spectrometry (LC-MS/MS) using an Acquity UPLC coupled to a Xevo TQ-S mass spectrometer (Waters, Milford, MA). Optimal extraction volumes for SPE were calculated using individual UV absorbance (λ = 300 nm) measurements to minimize matrix effects, and levels of 8-iso-PGF_2α_ were normalized to urinary creatinine concentrations as previously published [16].

### Microarray assessment

RNA from RNAlater-preserved IS cell pellet, BB, and BBr samples were extracted using Qiagen miRNeasy kit and amplified with Nugen ovation pico WTA kit (NuGen Technologies; San Carlos, CA). The cDNA was analysed using the Affymetrix HG-U133+PM microarray platform (Affymetrix, Santa Clara, CA). The primary raw data images (DAT files) were processed into numerical CEL files. CEL files were normalized, assessed for quality control to exclude technical outliers, and re-normalized using the robust multi-array (RMA) method. Batch effects from RNA processing sets were observed for the sputum and BBr datasets, with the batch effect adjusted in the data matrices using linear modeling of batch (as a random factor) and cohort in the ComBat R programme. For the sputum dataset, 3 subjects had duplicate samples, of which the mean of the log2 intensities, after RNA processing set batch adjustment, were used in the final analysis dataset. The limit of reliable quantification (LOD) was established from the inflection point of maximum variance with decreasing signal in a standard deviation vs. mean intensity plot across all probe sets and nonspecific probesets distribution. The cut-offs (number of probe sets included) were 5.5 (23496), 5.0 (18697), and 4.75 (21363) for sputum, BB, and BBr, respectively. The probe sets used in this study were as follow: NOX2 (203922_PM_s_at, 203923_PM_s_at, 217431_PM_x_at, 233538_PM_s_at); NOS1 (1560974_PM_s_at, 207309_PM_at, 207310_PM_s_at, 231916_PM_at, 239132_PM_at, 240911_PM_at); NOS2 (210037_PM_s_at); NOS3 (205581_PM_s_at); SOD1 (200642_PM_at); SOD2 (215078_PM_at, 215223_PM_s_at, 216841_PM_s_at, 221477_PM_s_at); SOD3 (205236_PM_x_at); CAT (201432_PM_at, 211922_PM_s_at, 215573_PM_at); GPX1 (200736_PM_s_at). Usually, probes are selected to represent genes and are designed to match particular mRNA transcripts, often based on deposited NCBI sequences. However, those sequences might be incorrect, partially inaccurate or incomplete due to different problems. Moreover a gene can have multiple splice variants [17]. Thus, multiple probe sets assigned to a common gene were studied separately in order to keep an unbiased reporting of all relevant probe sets. Public repository for microarray data are published in GEO website: GSE76262 (IS data); GSE76225 (BB data); GSE76226 (BBr data).

### Statistical analysis

Categorical data were summarized by counts and percentages; continuously distributed data exhibiting approximate symmetry of distribution were summarized using the mean (standard error; SE); continuously distributed data exhibiting skewness were summarized using the median (inter-quartile range; IQR). The latter all exhibited positive skewness and were log-transformed prior to parametric association testing. P-values were calculated by applying ANOVA to a generalised linear model. ANOVA was performed using logistic regression with adjustment for age and gender. All analyses were considered significant with a p-value of less than 5%. Acknowledging the modest sample size of the current exploratory study, adjustments for multiple-testing were not applied. Moreover, a negative result should be considered inconclusive. In order to characterise further some of the observed associations, rank correlation was assessed by calculation of Kendall’s Tau. A p-value was calculated in a test of the null hypothesis of zero correlation. Analyses of clinical and inflammatory data were performed using R version 2.15.2 (R Core Team, 2012).

## Results

### Clinical parameters

The clinical and inflammatory characteristics of all U-BIOPRED patients have been previously described [13]. Here, we compared the main clinical data between SAn and SAs/ex groups, as well as between all SA subjects when stratified by smoking status. *i*.*e*., CSA, ESA and NSA groups. These results are reported in S1–S4 Tables and Tables 1–4, respectively, for each subject subset (urine-8-iso-PGF_2α_; IS-transcriptomics; BB-transcriptomics; BBr-transcriptomics). In brief, the onset of asthma occurred later in SAs/ex than in SAn for urine-8-iso-PGF_2α_ (*p*< 0.001) and IS-transcriptimics (*p*< 0.031) subsets, although the subjects had a similar degree of airway obstruction (spirometry data). An older age at diagnosis was also observed in the CSA and/or ESA than NSA, but only in the urine-8-iso-PGF_2α_ subset (*p*< 0.001). Gastro-esophageal reflux disease (GERD) was increased in SAs/ex compared to SAn in the urine-8-iso-PGF_2α_ subset (*p* = 0.006), and this trend was also observed in the other subsets (*p*> 0.05). GERD was also increased in CSA for the urine-8-iso-PGF_2α_ subset (*p* = 0.009) compared to ESA and NSA. FEV_1_/FVC ratio was decreased in CSA than ESA and NSA with significant *p*-value in the urine-8-iso-PGF_2α_ subset (*p* = 0.033). FeNO levels were significantly lower in SAs/ex than SAn for those subjects for whom BB and BBr transcriptomic data was available, but this was not seen across all subsets. However, when compared SA smoking subgroups we observed that FeNO levels were significantly lower in CSA than ESA and NSA for all subject subsets. Regular OCS use was increased in ESA than CSA and NSA with significant p value in the urine-8-iso-PGF_2α_ (*p* = 0.002) and BBr transcriptomics (*p* = 0.009) subsets.

**Table 1:** Clinical and inflammatory characteristics of severe asthma non-, ex-, and current-smokers present in the Urinary 8-iso-PGF2α subset.

	NSA	ESA	CSA	*p*-value
**Subjects *n*.**	260	112	42	
**Age (yr)**	52 (42–61) [*n* = 260]	56 (50–62) [*n* = 112]	52.5 (46–58) [*n* = 42]	**<0.001**^§^
**Female**	171/260 (65.77%)	62/112 (55.36%)	24/42 (57.14%)	0.058
**Age at Diagnosis(yr)**	19.5 (7–37) [*n* = 252]	37.5 (20–48) [*n* = 110]	26.5 (7–42) [*n* = 42]	**<0.001**^§^
**Exacerbations (History)**	2 (1–3) [*n* = 259]	2 (1–3) [*n* = 112]	1 (0–4) [*n* = 42]	0.488
**Pack Years**	NA (NA-NA) [0]	8.43 (3–18) [*n* = 112]	20.15 (14–28) [*n* = 42]	**<0.001**^§^
**Allergic Rhinitis Diagnosed**	136/229 (59.39%)	46/106 (43.4%)	22/36 (61.11%)	**0.007**^§^
**Nasal Polyps Diagnosed**	79/241 (32.78%)	46/109 (42.2%)	9/36 (25%)	0.089
**GERD Diagnosed**	108/240 (45%)	64/106 (60.38%)	23/35 (65.71%)	**0.009**^§^
**FEV**_**1**_**% pred**	67.74 (51–85) [*n* = 257]	65.67 (52–82) [*n* = 112]	64.48 (50–75) [*n* = 42]	0.561
**FVC % pred**	86.97 (73–101) [*n* = 257]	90.25 (77–102) [*n* = 112]	86.88 (77–97) [*n* = 42]	0.104
**FEV**_**1**_**/FVC ratio**	0.65± 0.01 [*n* = 257]	0.61± 0.01 [*n* = 112]	0.6± 0.02 [*n* = 42]	**0.033**^§^
**Exhaled NO**	27 (16–48) [*n* = 240]	25 (15–48) [*n* = 106]	16 (10–31) [*n* = 41]	**<0.001**^§^
**Sputum Eosinophils**	15 (2–84) [*n* = 105]	20 (2–85) [*n* = 55]	13 (4–36) [*n* = 21]	0.585
**Sputum Neutrophils**	276 (160–400) [*n* = 105]	281 (200–382) [*n* = 55]	292 (203–343) [*n* = 21]	0.28
**Sputum Eosinophils (%)**	2.86 (0–18) [*n* = 105]	3.81 (1–17) [*n* = 55]	2.47 (1–7) [*n* = 21]	0.572
**Sputum Neutrophils (%)**	53.98 (32–75) [*n* = 105]	55.1 (44–72) [*n* = 55]	55.94 (35–64) [*n* = 21]	0.226
**Mean ACQ with ACQ7**	2.71 (2–4) [*n* = 236]	2.43 (2–3) [*n* = 95]	2.79 (2–3) [*n* = 38]	0.135
**Regular ICS or ICS/LABA Use**	259/260 (99.62%)	112/112 (100%)	42/42 (100%)	0.997
**Regular Oral Corticosteroids**	117/250 (46.8%)	53/103 (51.46%)	8/40 (20%)	**0.002**^§^

Data are presented as mean±SE [*n*], median (interquartile range) [*n*] or *n*/N (%), unless otherwise stated. ACQ: Asthma Control Questionnaire; CSA: current smokers with severe atshma; ESA: ex-smokers with severe asthma; FEV_1_: forced expiratory volume in 1 second; FVC: forced vital capacity; GERD: gastro-esophageal reflux disease; ICS: inhaled corticosteroids; LABA: long-acting β_2_-agonist; NSA: non-smokers with severe asthma.

^§^ significant *p* value

**Table 2:** Clinical and inflammatory characteristics of severe asthma non-, ex-, and current-smokers present in the induced sputum-transcriptomic subset.

	NSA	ESA	CSA	*p*-value
**Subjects *n*.**	47	29	8	
**Age (yr)**	53 (44–60) [*n* = 47]	56 (52–62) [*n* = 29]	46.5 (45–55) [*n* = 8]	0.073
**Female**	28/47 (59.57%)	17/29 (58.62%)	4/8 (50%)	0.613
**Age at Diagnosis (yr)**	17 (5–38) [*n* = 47]	38.5 (26–49) [*n* = 28]	24 (7–33) [*n* = 8]	0.563
**Exacerbations (History)**	2 (1–3) [*n* = 47]	2 (1–3) [*n* = 29]	1.5 (1–4) [*n* = 8]	0.593
**Pack Years**	NA (NA_NA) [*n* = 0]	7 (2–15) [*n* = 29]	18.25 (13–23) [*n* = 8]	**0.034**^§^
**Allergic Rhinitis Diagnosed**	19/39 (48.72%)	10/28 (35.71%)	2/7 (28.57%)	0.291
**Nasal Polyps Diagnosed**	16/45 (35.56%)	12/29 (41.38%)	2/7 (28.57%)	0.614
**GERD Diagnosed**	17/45 (37.78%)	17/28 (60.71%)	4/6 (66.67%)	0.059
**FEV**_**1**_**% pred**	59.93 (45–74) [*n* = 47]	63.93 (55–75) [*n* = 29]	73.49 (70–76) [*n* = 8]	0.082
**FVC % pred**	85.29 (73–98) [*n* = 47]	93.66 (81–108) [*n* = 29]	99.07 (86–105) [*n* = 8]	**0.018**^§^
**FEV**_**1**_**/FVC ratio**	0.57± 0.02 [*n* = 47]	0.58± 0.02 [*n* = 29]	0.61± 0.03 [*n* = 8]	0.416
**Exhaled NO**	26 (19–49) [*n* = 45]	28.5 (15–53) [*n* = 28]	10.75 (8–18) [*n* = 8]	**0.007**^§^
**Sputum Eosinophils**	15 (2–69) [*n* = 47]	29 (2–89) [*n* = 29]	17 (2–41) [*n* = 8]	0.505
**Sputum Neutrophils**	327 (192–434) [*n* = 47]	281 (235–381) [*n* = 29]	271 (234–328) [*n* = 8]	0.414
**Sputum Eosinophils (%)**	2.86 (0–14) [*n* = 47]	5.66 (0–19) [*n* = 29]	3.23 (0–8) [*n* = 8]	0.575
**Sputum Neutrophils (%)**	68.22 (35–84) [*n* = 47]	55.15 (47–74) [*n* = 29]	53.55 (44–64) [*n* = 8]	0.369
**Mean ACQ with ACQ7**	2.71 (1–4) [*n* = 44]	2.14 (2–3) [*n* = 26]	2.29 (2–4) [*n* = 7]	0.609
**Regular ICS or ICS/LABA Use**	47/47 (100%)	29/29 (100%)	8/8 (100%)	1
**Regular Oral Corticosteroids**	19/46 (41.3%)	16/27 (59.26%)	3/7 (42.86%)	0.141

Data are presented as mean±SE [*n*], median (interquartile range) [*n*] or *n*/N (%), unless otherwise stated. ACQ: Asthma Control Questionnaire; CSA: current smokers with severe asthma; ESA: ex-smokers with severe asthma; FEV_1_: forced expiratory volume in 1 second; FVC: forced vital capacity; GERD: gastro-esophageal reflux disease; ICS: inhaled corticosteroids; LABA: long-acting β_2_-agonist; NSA: non-smokers with severe asthm.

^§^ significant *p* value

**Table 3:** Clinical and inflammatory characteristics of severe asthma non-, ex-, and current-smokers present in the bronchial biopsy-transcriptomic subset.

	NSA	ESA	CSA	*p*-value
**Subjects *n*.**	34	12	7	
**Age (yr)**	51.5 (43–60) [*n* = 34]	54.5 (43–62) [*n* = 12]	52 (46–55) [*n* = 7]	0.779
**Female**	21/34 (61.76%)	2/12 (16.67%)	5/7 (71.43%)	**0.014**^§^
**Age at Diagnosis(yr)**	10 (2–40) [*n* = 33]	5.5 (2–38) [*n* = 12]	33 (18–44) [*n* = 7]	0.429
**Exacerbations (History)**	2 (0–4) [*n* = 33]	2.5 (1–3) [*n* = 12]	4 (2–5) [*n* = 7]	0.078
**Pack Years**	NA (NA_NA) [*n* = 0]	5.25 (2–25) [*n* = 12]	20.5 (17–24) [*n* = 7]	0.067
**Allergic Rhinitis Diagnosed**	19/31 (61.29%)	6/12 (50%)	2/7 (28.57%)	0.132
**Nasal Polyps Diagnosed**	10/32 (31.25%)	8/12 (66.67%)	1/7 (14.29%)	**0.04**^§^
**GERD Diagnosed**	16/33 (48.48%)	9/12 (75%)	4/6 (66.67%)	0.123
**FEV**_**1**_**% pred**	74.17 (54–89) [*n* = 34]	69.35 (57–77) [*n* = 12]	68.7 (55–72) [*n* = 7]	0.405
**FVC % pred**	89.19 (78–103) [*n* = 34]	87.15 (75–97) [*n* = 12]	93.8 (91–100) [*n* = 7]	0.443
**FEV**_**1**_**/FVC ratio**	0.67± 0.02 [*n* = 34]	0.65± 0.03 [*n* = 12]	0.58± 0.03 [*n* = 7]	0.081
**Exhaled NO**	30 (20–46) [*n* = 29]	23 (20–50) [*n* = 12]	8 (7–23) [*n* = 7]	**0.005**^§^
**Sputum Eosinophils**	20 (6–84) [*n* = 18]	2.5 (2–9) [*n* = 4]	6.5 (2–14) [*n* = 4]	0.176
**Sputum Neutrophils**	254 (215–319) [*n* = 18]	364 (216–470) [*n* = 4]	260 (154–347) [*n* = 4]	0.105
**Sputum Eosinophils (%)**	3.62 (1–16) [*n* = 18]	0.53 (0–2) [*n* = 4]	1.3 (0–3) [*n* = 4]	0.134
**Sputum Neutrophils (%)**	51.47 (41–56) [*n* = 18]	66.66 (40–88) [*n* = 4]	49.23 (27–69) [*n* = 4]	0.119
**Mean ACQ with ACQ7**	2 (1–3) [*n* = 29]	2.29 (1–4) [*n* = 10]	3 (2–4) [*n* = 5]	0.092
**Regular ICS or ICS/LABA Use**	34/34 (100%)	12/12 (100%)	7/7 (100%)	1
**Regular Oral Corticosteroids**	14/32 (43.75%)	6/11 (54.55%)	2/7 (28.57%)	0.465

Data are presented as mean±SE [*n*], median (interquartile range) [*n*] or *n*/N (%), unless otherwise stated. ACQ: Asthma Control Questionnaire; CSA: current smokers with severe asthma; ESA: ex-smokers with severe asthma; FEV_1_: forced expiratory volume in 1 second; FVC: forced vital capacity; GERD: gastro-esophageal reflux disease; ICS: inhaled corticosteroids; LABA: long-acting β_2_-agonist; NSA: non-smokers with severe asthm.

^§^ significant *p* value

**Table 4:** Clinical and inflammatory characteristics of severe asthma non-, ex-, and current-smokers present in the bronchial brushing-transcriptomic subset.

	NSA	ESA	CSA	*p*-value
**Subjects *n*.**	40	21	6	
**Age (yr)**	51 (40–59) [*n* = 40]	53 (46–62) [*n* = 21]	53.5 (44–55) [*n* = 6]	0.413
**Female**	22/40 (55%)	6/21 (28.57%)	4/6 (66.67%)	0.053
**Age at Diagnosis(yr)**	15.5 (5–42) [*n* = 38]	24 (5–39) [*n* = 21]	31.5 (13–40) [*n* = 6]	0.563
**Exacerbations (History)**	2 (1–4) [*n* = 39]	2 (1–3) [*n* = 21]	3.5 (2–5) [*n* = 6]	0.283
**Pack Years**	NA (NA_NA) [*n* = 0]	5.5 (3–22) [*n* = 21]	19.5 (17–23) [*n* = 6]	0.122
**Allergic Rhinitis Diagnosed**	21/35 (60%)	11/21 (52.38%)	1/6 (16.67%)	0.079
**Nasal Polyps Diagnosed**	12/35 (34.29%)	12/20 (60%)	1/6 (16.67%)	0.068
**GERD Diagnosed**	21/37 (56.76%)	13/21 (61.9%)	4/5 (80%)	0.339
**FEV**_**1**_**% pred**	79.33 (57–93) [*n* = 40]	65.66 (55–74) [*n* = 21]	69.48 (61–73) [*n* = 6]	0.072
**FVC % pred**	94.27 (77–104) [*n* = 40]	85.78 (76–94) [*n* = 21]	95.53 (93–101) [*n* = 6]	0.188
**FEV**_**1**_**/FVC ratio**	0.69± 0.02 [*n* = 40]	0.62± 0.02 [*n* = 21]	0.59± 0.04 [*n* = 6]	0.054
**Exhaled NO**	31.25 (19–54) [*n* = 36]	23.5 (20–63) [*n* = 20]	7.75 (7–13) [*n* = 6]	**0.001**^§^
**Sputum Eosinophils**	20 (2–94) [*n* = 16]	3 (2–26) [*n* = 9]	3 (2–6) [*n* = 3]	0.216
**Sputum Neutrophils**	235 (208–312) [*n* = 16]	263 (183–454) [*n* = 9]	317 (260–378) [*n* = 3]	0.556
**Sputum Eosinophils (%)**	3.62 (0–18) [*n* = 16]	0.71 (0–5) [*n* = 9]	0.6 (0–1) [*n* = 3]	0.183
**Sputum Neutrophils (%)**	49.23 (39–60) [*n* = 16]	46.88 (32–86) [*n* = 9]	63.4 (49–75) [*n* = 3]	0.543
**Mean ACQ with ACQ7**	2 (1–3) [*n* = 31]	2.29 (1–3) [*n* = 16]	3 (2–4) [*n* = 5]	0.081
**Regular ICS or ICS/LABA Use**	40/40 (100%)	21/21 (100%)	6/6 (100%)	1
**Regular Oral Corticosteroids**	13/37 (35.14%)	14/19 (73.68%)	1/6 (16.67%)	**0.009**^§^

Data are presented as mean±SE [*n*], median (interquartile range) [*n*] or *n*/N (%), unless otherwise stated. ACQ: Asthma Control Questionnaire; CSA: current smokers with severe asthma; ESA: ex-smokers with severe asthma; FEV_1_: forced expiratory volume in 1 second; FVC: forced vital capacity; GERD: gastro-esophageal reflux disease; ICS: inhaled corticosteroids; LABA: long-acting β_2_-agonist; NSA: non-smokers with severe asthm.

^§^ significant *p* value

### Lipid peroxidation

**Fig 1:**
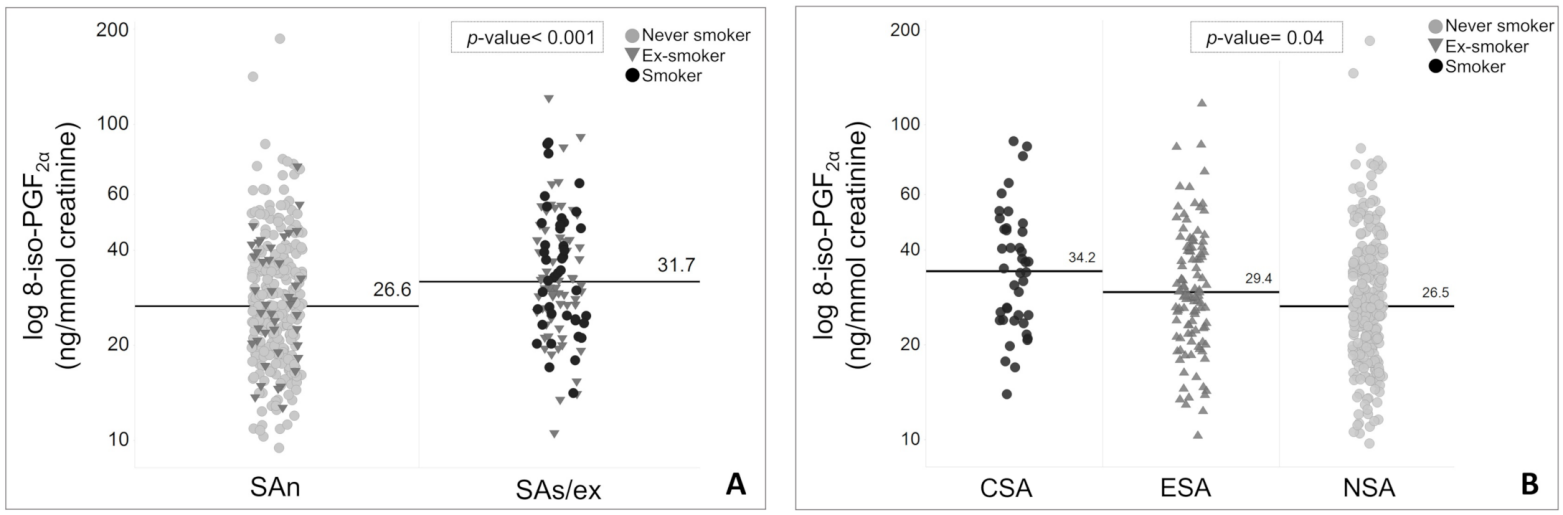
(**A)** Comparison of 8-iso-PGF2α in urine between SAn and SAs/ex. (**B)** Comparison of urinary 8-iso-PGF2α between severe asthma smoking subgroups. CSA: current smokers with severe asthma; ESA: ex-smokers with severe asthma; NSA: non smokers with severe asthma; SAn: Severe Asthma non smokers; SAs/ex: Severe Asthma smokers/ex-smokers.

We analysed 8-iso-PGF_2α_, a specific biomarker of lipid peroxidation, in spot urine samples. The median (IQR) concentration of 8-iso-PGF_2α_ was significantly increased in the urine of SAs/ex compared to SAn, 31.7 (24.5–44.7) *vs*. 26.6 (19.6–36.6) ng/mmol creatinine respectively (FC = 1.19; *p*< 0.001) (Fig 1A), Furthermore, we assessed levels of 8-iso-PGF_2α_ taking into consideration smoking status within the two combined SA cohorts (SAn+SAs/ex). A significant difference was observed between the smoking subgroups (*p* = 0.004) with increased median (IQR) concentration of urinary 8-iso-PGF_2α_ in CSA, 34.25 (24.4–47.7), *vs* ESA, 29.4 (22.3–40.5), and NSA, 26.5 (19.6–16.6) ng/mmol creatinine (Fig 1B).

### mRNA expression of pro-/anti-oxidant enzymes

All the results about mRNA expression of pro-/antioxidant enzymes are reported in Table 5. Sputum mRNA levels of the pro-oxidant enzymes NOX2 and NOS3 were examined. The mRNA levels of NOX2 were increased in SAs/ex compared to SAn. In particular the probe sets 203922_PM_s_at (FC = 1.05 *p* = 0.006), 203923_PM_s_at (FC = 1.06; *p* = 0.003), 233538_PM_s_at (FC = 1.06; *p* = 0.014) were over-expressed in SAs/ex. The NOX2 probe set 217431_PM_x_at is less abundant than the other probe sets, and was not significantly different between SAn and SAs/ex (FC = 1.00; *p* = 0.950). The expression of NOS3 mRNA in IS was similar between SAs/ex and SAn, while the levels of NOS1, NOS2 probe sets, together with the anti-oxidative SOD3, were below the limit of detection (LOD). CAT mRNA levels were over-expressed in IS of SAs/ex compared to SAn for the probe set 201432_PM_at (FC = 1.07; *p* = 0.028), but the probe set 211922_PM_s_at did not show a significant difference (*p* = 0.359). By contrast, IS mRNA expression of SOD1, SOD2, SOD3 and GPX1 were not statistically different between SAn and SAs/ex.

**Table 5:** Comparison of pro-/anti-oxidant enzyme mRNA expression in induced sputum, bronchial biopsies and bronchial brushings between severe asthma cohorts.

Gene symbol	Probe set ID	SAn mean	SAs/ex mean	Fold Change	*p* value
***Induced Sputum (SAn n = 61; SAs/ex n = 23)***: LOD *= 5*.*5 log2 intensity*					
**NOX2**	203922_PM_s_at	9.89	10.35	1.05	**0.006**
	203923_PM_s_at	9.03	9.59	1.06	**0.003**
	217431_PM_x_at	5.96	5.97	1	0.950
	233538_PM_s_at	6.57	6.96	1.06	**0.014**
**NOS3**	205581_PM_s_at	6.32	6.19	-1.02	0.115
**SOD1**	200642_PM_at	8.37	8.69	1.04	0.094
**SOD2**	215078_PM_at	9.93	9.62	-1.03	0.225
	215223_PM_s_at	11.65	11.46	-1.02	0.303
	216841_PM_s_at	11.40	11.20	-1.02	0.098
	221477_PM_s_at	11.26	10.99	-1.02	0.121
**CAT**	201432_PM_at	8.41	9.01	1.07	**0.028**
	211922_PM_s_at	7.81	7.97	1.02	0.359
**GPX1**	200736_PM_s_at	9.57	9.74	1.02	0.385
***Bronchial Biopsy (SAn n = 40; SAs/ex n = 13)***: *LOD = 5 log2 intensity*					
**SOD1**	200642_PM_at	8.36	8.30	-1.01	0.253
**SOD2**	215223_PM_s_at	7.00	7.03	1.00	0.667
	216841_PM_s_at	6.35	6.10	-1.04	0.075
	221477_PM_s_at	6.28	6.11	-1.03	0.148
**SOD3**	205236_PM_x_at	6.77	7.07	1.04	0.161
**CAT**	201432_PM_at	6.84	6.89	1.01	0.941
	211922_PM_s_at	5.73	5.68	-1.01	0.558
**GPX1**	200736_PM_s_at	7.04	7.19	1.02	0.437
***Bronchial Brushing (SAn n = 49; SAs/ex n = 18)***: *LOD = 4*.*5 log2 intensity*					
**NOS2**	210037_PM_s_at	5.36	4.87	-1.10	**0.029**
**NOS3**	205581_PM_s_at	4.97	4.88	-1.02	0.310
**SOD1**	200642_PM_at	9.03	8.99	-1.00	0.802
**SOD2**	215223_PM_s_at	7.58	7.25	-1.05	0.176
	216841_PM_s_at	6.90	6.56	-1.05	0.165
	221477_PM_s_at	6.84	6.60	-1.04	0.206
**CAT**	201432_PM_at	7.47	7.39	-1.01	0.620
	211922_PM_s_at	6.20	6.24	1.01	0.681
**GPX1**	200736_PM_s_at	7.56	7.58	1.00	0.960

*P*-values were calculated by applying ANOVA with adjustment for age and gender. LOD: limit of detection; NOX2: NADPH oxidase 2; NOS2/3: nitric oxide synthase 2/3; SOD1/2/3: superoxide dismutase 1/2/3; CAT: catalase; GPX1: glutathione peroxidase 1; SAn: severe asthma non smokers; SAs/ex: severe asthma smokers/ex-smokers.

All of the pro-oxidant enzymes were excluded in BB analyses because their expression levels were below the LOD. The expression of all antioxidant enzymes in BB was similar among SAn and SAs/ex.

NOS2 mRNA levels were significantly decreased in BBr of SAs/ex compared to SAn (FC = -1.10; *p* = 0.029). BBr NOS3 mRNA expression was similar between SAn and SAs/ex (FC = -1.01; *p* = 0.310). In addition, the mRNA expression levels of anti-oxidant enzymes in BBr were similar among the two study groups. NOX2, NOS1 and SOD3 were excluded from the analysis in BBr subset because their mRNA expression levels were below the LOD.

When stratification was made based on smoking status a low number of subjects were available for which oxidant/anti-oxidant enzyme mRNA expression data could be used with sufficient statistical power and therefore this analysis was not performed.

### Correlation analysis

**Fig 2:**
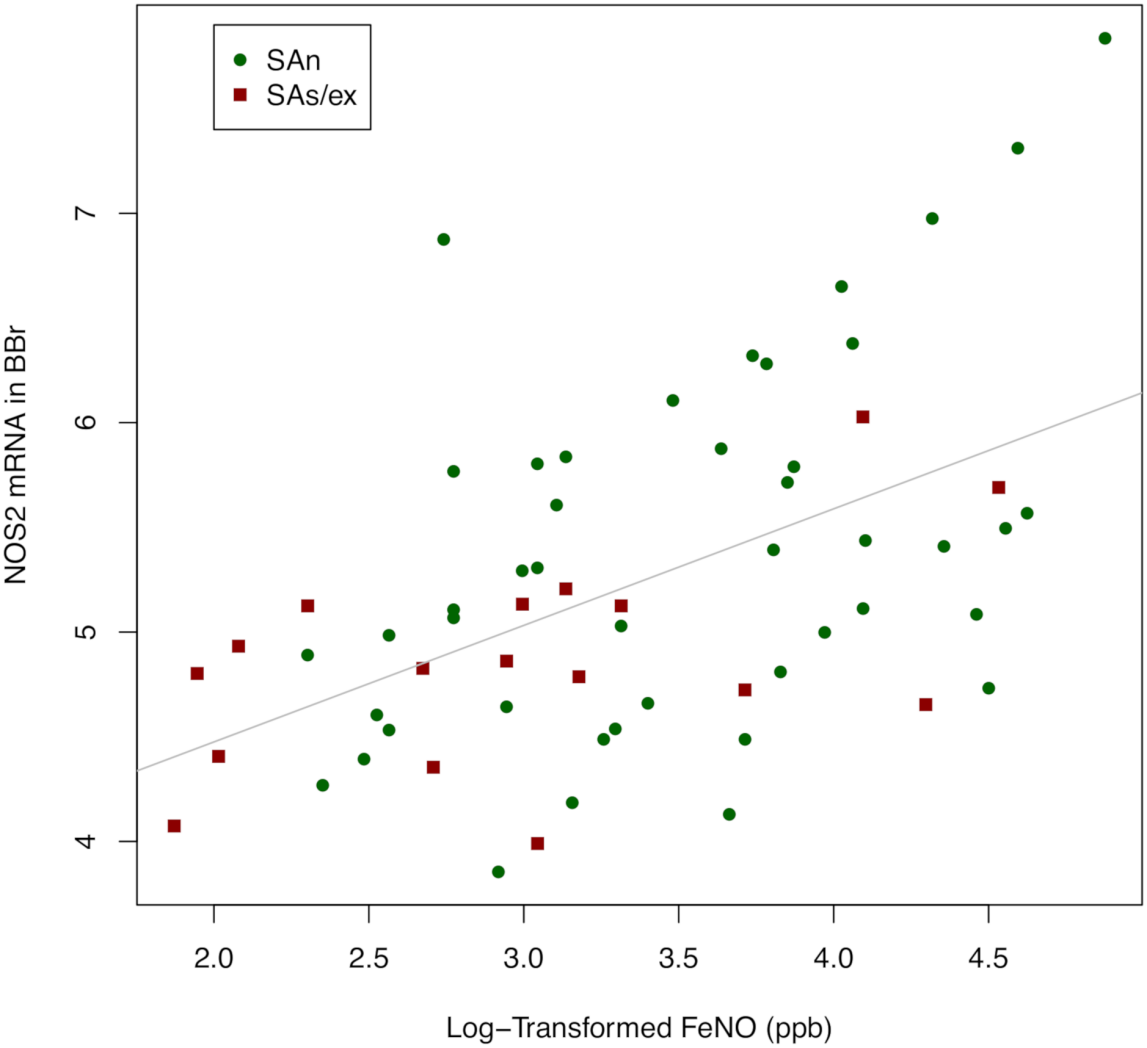
Scatter plot of the relationship between NOS2 expression in bronchial brushing and FeNO. NOS2 expression in bronchial brushing were strongly correlated to FeNO. Kendall’s Tau = 0.535, *p*< 0.001, (*n* = 62). FeNO (ppb) were log2-transformed. SAn: Severe Asthma non smokers; SAs/ex: Severe Asthma smokers/ex-smokers.

Two NOX2 probe sets in sputum correlated significantly with macrophage numbers (203922_PM_s_at Kendall’s Tau = 0.49, p< 0.001; 203923_PM_s_at Kendall’s Tau = 0.45 *p*< 0.001) and percentages (203922_PM_s_at Kendall’s Tau = 0.48, p< 0.001; 203923_PM_s_at Kendall’s Tau = 0.43 p< 0.001). Inversely correlations were observed between three NOX2 probe sets and eosinophil numbers (203922_PM_s_at Kendall’s Tau = -0.19, *p* = 0.01; 203923_PM_s_at Kendall’s Tau = -0.24, *p* = 0.002; 233538_PM_s_at Kendall’s Tau = -0.22, *p* = 0.004), and percentages (203922_PM_s_at Kendall’s Tau = -0.20, p = 0.008; 203923_PM_s_at Kendall’s Tau = -0.24, p = 0.001; 233538_PM_s_at Kendall’s Tau = -0.22, *p* = 0.004). One NOX2 probe set in sputum inversely correlated with neutrophil numbers (203922_PM_s_at Kendall’s Tau = -0.18, p = 0.014), while two NOX2 probe sets inversely correlated with neutrophil percentages (203922_PM_s_at Kendall’s Tau = -0.18, p = 0.014; 203923_PM_s_at Kendall’s Tau = -0.15, p = 0.038) However, there was no correlation between NOX2 mRNA in sputum and 8-iso-PGF_2α_ in urine (all the four probe sets had *p*> 0.10). Moreover, a strong correlation between NOS2 in BBr and FeNO was observed with a Kendall’s Tau = 0.535 (*p*< 0.001) (Fig 2). The correlation analysis between NOS2 in BBr and FeNO was also performed for each smoking status group (*i*.*e*., NSA, ESA, CSA): where we observed a significant correlation in 36 NSA (Kendall’s Tau = 0.551; *p*< 0.001); in 20 ESA (Kendall’s Tau = 0.394; *p* = 0.085); the number of CSA subjects was six and therefore correlation analysis was considered not appropriate.

## Discussion

We used data from the U-BIOPRED severe asthma cohorts to assess the effect of cigarette smoke on oxidative stress markers in severe asthma subjects with a significat smoking history. We found an increased systemic oxidative stress in SAs/ex, and in particular among severe asthma current smokers, who exhibited the highest concentration of urinary 8-iso-PGF_2α_. In sputum, NOX2 mRNA expression was increased in SAs/ex compared to SAn, while NOS2 mRNA expression was decresead in bronchial brushing of SAs/ex. Moreover, levels of FeNO were decresed in severe asthma current smokers, and was correlated with NOS2 mRNA expression in bronchial brushing.

Tobacco smoke is one of the major environmental sources of oxidative stress and can lead to greater lipid peroxidation. It has been shown that 8-iso-PGF_2α_ levels are elevated with asthma severity [11] and further enhanced during acute exacerbations [18] and allergen challenge in asthmatics [11]. Our data provide additional support for the findings in other studies, showing the ability of cigarette smoke to increase isoprostane levels, by the increased level of urinary 8-iso-PGF_2α_ in SAs/ex, and in particular, in the current smokers subgroup [19,20]. However, our data extended the previous findings showing the incresase of this oxidative stress biomarker in smokers with severe asthma. F2 isoprostanes have a potent smooth muscle and vascular constrictive action which increases airway hyperresponsiveness and obstruction, and induces plasma exudation and inflammation [21]. Thus, the increased formation of 8-iso-PGF_2α_ in smoking asthmatics, as induced by cigarette smoke, may enhance disease progression and asthma symptoms.

The NOX2 isoform is primarily present in macrophages, neutrophils and eosinophils and is most highly abundant in IS. NOX2 activity is normally required for phagocyte respiratory burst and regulation of cell signaling [22,23]. Furthermore, the enzyme NOX2 is activated by cigarette smoke through the phosphorylation of c-Src [24], a tyrosine kinase protein, leading to a higher production of ROS [24,25]. In our study, NOX2 mRNA is over-expressed in sputum of SAs/ex compared to SAn, supporting the hypothesis that exposure to smoke in asthmatic subjects is able to amplify NOX2 mRNA expression with a consequent elevation of O2^•-^ production. However, we could not establish a significant correlation between NOX2 over-expression in IS and urinary 8-iso-PGF_2α_. Certainly, NOX2 is just one of the several factors contributing to the increase of oxidative stress. The positive correlation of NOX2 mRNA with macrophages in sputum and the inverse relationship with phagocytes may highlight the importance of distinct cellular phenotypes in regulating the inflammatory process in severe asthmatics who smoke [26,27]. This area deserves further investigation.

NOS enzymes are important pro-oxidants producing NO, an endogenous regulator involved in homeostatic and immunological functions with a role in asthma pathogenesis [9]. We observed no differences in mRNA expression of constitutive NOS3 between SAn and SAs/ex, which suggests that cigarette smoke has a weak relation to NOS3 expression, if any. NOS2 is the inducible isoform (iNOS), which produces high levels of NO and its activity persists for many days after induction [28,29], leading to cell death and tissue damage [28]. This enzyme is mainly expressed in lung epithelium [29], so it is mainly detectable in BBr. NOS2 mRNA expression is induced by pro-inflammatory cytokines [9,30], and is increased in asthma in proportion to the severity of the disease [31], and in particular by allergen provocation [32]. In this study, levels of NOS2 mRNA from BBr of SAs/ex was lower than that of SAn. Furthermore, we demonstrated a strong correlation between FeNO levels and BBr NOS2 mRNA expression in SA cohorts, as also have been reported previously [33]. Another study has shown that NO generated by NOS2 is able to attenuate its own expression through the negative regulation of NF-κB [34]. Therefore, we hypothesise that active exposure to cigarette smoke can lead to inhibition of NOS2 mRNA expression in a negative feedback manner.

Evidence exists of lower FeNO levels in asthmatic smokers [35,36], which has also been shown in this U-BIOPRED adult SAs/ex cohort [13], indicating a possible effect of active smoking on levels of exhaled NO. Further analysis of FeNO, in relation to the cigarette smoking status of patients (current-, ex- and never-smokers), showed decreased FeNO levels in current smokers confirming the effect of active smoking on exhaled NO [36]. Despite the fact that superoxide concentration is known to be high in cigarette smoke, and thereby it can enhance the reduction of FeNO by reacting with available NO, we demonstrated a significant correlation between NOS2 in bronchial brushings and FeNO in severe asthmatic subjects. Moreover, the peroxynitrite product of this reaction is a very strong oxidant species [8–10]. Thus, knowledge of current smoking status is important when using FeNO measurements in assessing asthma control and severity.

The anti-oxidant enzymes—SODs, CAT and GPX1 —catalyze reactions to neutralize oxidative toxic intermediates. SODs are the primary enzymes able to dismantle superoxide anion to form H_2_O_2_. Whereas, CAT and GPX1 are key antioxidant enzymes for the degradation of reactive H_2_O_2_ to H_2_O and O_2_ [10]. We observed no significant differences in SOD1, SOD2 and SOD3 mRNA expression, as well as for CAT and GPX1 mRNA expression, in airways of SAn and SAs/ex. To our knowledge, there are no reports concerning antioxidant enzyme status in severe asthma smokers. However, some evidences relating to expression of SODs, CAT and GPX1 enzymes in relation to asthma or cigarette smoke were found in the literature. Several studies reported that SOD1 has low activity and expression in asthmatic airways [10,37] and, in addiction, SOD2 was found to be inactivated and down regulated in asthmatic patients [37–39]. Furthermore, the expression of SOD3 seems to be decreased *in vitro* by TNF-α, TGF-β and IL1-α, while it is enhanced by IFN-γ [10,40]. Cigarette smoke enhanced the expression [41] and activity [42] of SODs in rat airways. Conversely, the levels of SOD were found increased in blood and saliva of subjects who smoke [43], and prolonged cigarette smoke exposure was found to increase the mRNA level of SOD2 in human bronchial epithelial cells [44]. Therefore, it is conceivable that whilst SOD mRNA expression is decreased in SA, smoking enhances SOD mRNA expression, resulting in a balance between the two actions and overall no difference between SAn and SAs/ex. Catalase (protein and mRNA levels) was previously reported as decreased in the bronchiolar epithelium of smokers with COPD [45]. Furthermore, the activity of CAT and GPX was previously found reduced in asthmatic patients [10]. Further studies are required to determine whether the decreased expression reported by Betsuyaku et al. [45] was a result of smoking or of COPD.

### Limitations of the study

Due to the explorative nature of this study, there are several limitations. One limitation is the absence of a healthy smoker and a healthy non-smokers control groups, which do not allow us to establish for sure the influence of cigarette smoking and/or severe asthma. Moreover, the measurement of whole body excretion of 8-iso-PGF_2α_ in urine cannot determine the source (airways or systemic source). Given that the patients included in this study have the same degree of asthma severity it is however likely that the observed changes are predominantly due to smoking.

Few patients provided samples from each compartment within the U-BIOPRED study. Particularly, the number of SAs/ex samples is low for the sputum, BB and BBr transcriptomics set, thus we cannot didived SAs/ex group into current and ex-smokers for the mRNA expression analysis.

We evaluated the mRNA expression of pro-/anti-oxidant enzymes, but the mRNA levels do not necessarily correlate with the activity of the corresponding enzymes or their products’ concentration. Moreover, mRNA-expression of several enzymes could not be accurately assessed due to their low expression levels on the microarray. Although we report significant differences in oxidant gene expression of SAs/ex, the changes are small and other mechanisms driving asthma severity in these patients may be present. Independent replications of these findings are warranted.

## Conclusions

In conclusion, our results indicate that severe asthmatics who smoke have evidence for increased systemic oxidative stress. The increased mRNA expression of NOX2 in the airway lumen could contribute to this phenomenon. However, this is the first study in severe asthma showing a clear relationship between cigarette smoking and reduced NOS2 expression together with lower levels of FeNO. Future studies are needed to investigate this complex mechanism in the framework of smoking related to severe asthma.

## Supporting information

S1 Table Clinical and inflammatory characteristics of subjects present in the urinary 8-iso-PGF2α subset. (DOCX)

S2 Table Clinical and inflammatory characteristics of subjects present in the induced sputum subset. (DOCX)

S3 Table Clinical and inflammatory characteristics of subjects present in the bronchial biopsy subset. (DOCX)

S4 Table Clinical and inflammatory characteristics of subjects present in the bronchial brushing subset. (DOCX)

S5 Table U-BIOPRED Consortium Information. (DOCX)

S1 File Materials and methods. (DOCX)

S2 File Clinical and inflammatory_ Data. (XLSX)

S3 File mRNA expression_IS_Data. (XLSX)

S4 File mRNA expression_BB_Data. (XLSX)

S5 File mRNA expression_BBr_Data. (XLSX)

## Abbreviations

8-iso-PGF2α: 8-Isoprostaglandin F2α
BB: Bronchial Biopsy
BBr: Bronchial Brushing
CAT: catalase
CSA: current smokers with severe asthma
ESA: ex-smokers with severe asthma
FC: Fold change
GPX1: glutathione peroxidase 1
IS: Induced Sputum
LOD: limit of detection
NOS: nitric oxide synthase
NOX2: NADPH oxidase 2
NSA: non smokers with severe asthma
RNS: reactive nitrogen species
ROS: reactive oxygen species
SA: severe asthma/asthmatic
SAn: severe asthma non smokers
SAs/ex: severe asthma smokers/ex-smokers
SOD: superoxide dismutase
U-BIOPRED: Unbiased BIOmarkers for the PREDiction of Respiratory Disease Outcomes
