# Supplementary material for: Enhanced oxidative stress in smoking and ex-smoking severe asthma in the U-BIOPRED cohort
Source: PLoS One. 2018 Sep 21;13(9):e0203874. doi: 10.1371/journal.pone.0203874 (PMC6150501; doi:10.1371/journal.pone.0203874)
Supplement: S1 Table — (DOCX) [file pone.0203874.s001.docx]

Table S1. Clinical and inflammatory characteristics of subjects present in the urinary 8-iso-PGF2α subset.

|  | SAn | SAs/ex | *p*-value | |
| --- | --- | --- | --- | --- |
| Subjects *n*. | 302 | 109 | |  |
| Age (yr) | 53 (43-62) [*n*=302] | 55 (48-61) [*n*=109] | | **0.016** |
| Female | 201/302 (65.9%) | 56/109 (51.38%) | | **0.008** |
| Age at Diagnosis(yr) | 20.50 (7-38) [*n*=296] | 37.50 (20-48) [*n*=108] | | **<0.001** |
| Exacerbations (History) | 2 (1-3) [*n*=302] | 2 (1-4) [*n*=109] | | 0.767 |
| Pack Years | 2 (1-4) [*n*=45] | 17.25 (10-26) [*n*=109] | | **<0.001** |
| Allergic Rhinitis Diagnosed | 160/271 (59.04%) | 44/100 (44%) | | **0.010** |
| Nasal Polyps Diagnosed | 100/286 (34.97%) | 34/100 (34%) | | 0.861 |
| GERD Diagnosed | 133/283 (47%) | 62/98 (63.27%) | | **0.006** |
| FEV_1_ % pred | 67.28±1.28 [*n*=302] | 67.39±1.85 [*n*=109] | | 0.647 |
| FVC % pred | 87.19±1.13 [*n*=302] | 89.89±1.74 [*n*=109] | | 0.163 |
| FEV_1_/FVC ratio | 0.64±0.01 [*n*=302] | 0.61±0.01 [*n*=109] | | 0.098 |
| Exhaled NO | 26.50 (16-47) [*n*=284] | 23.00 (12-43) [*n*=103] | | 0.091 |
| Sputum Eosinophils | 14.00 (2-85) [*n*=128] | 21.00 (4-70) [*n*=53] | | 0.680 |
| Sputum Neutrophils | 274 (169-402) [*n*=128] | 296 (199-354) [*n*=53] | | 0.346 |
| Sputum Eosinophils (%) | 2.75 (0-19) [*n*=128] | 4.13 (1-14) [*n*=53] | | 0.959 |
| Sputum Neutrophils (%) | 53.69 (34-75) [*n*=128] | 55.15 (35-65) [*n*=53] | | 0.433 |
| Mean ACQ with ACQ7 | 2.71 (2-4) [*n*=274] | 2.57 (2-3) [*n*=95] | | 0.951 |
| Regular ICS or ICS/LABA Use | 301/302 (99.67%) | 109/109 (100%) | | 0.997 |
| Regular Oral Corticosteroids | 133/291 (45.7%) | 45/102 (44.12%) | | 0.782 |
| Data are presented as mean±SE [*n*], median (interquartile range) [*n*] or *n*/N (%), unless otherwise stated. ACQ: Asthma Control Questionnaire; FEV_1_: forced expiratory volume in 1 second; FVC: forced vital capacity; GERD: gastro-esophageal reflux disease; ICS: inhaled corticosteroids; LABA: long-acting β_2_-agonist; SAn: severe asthma non-smokers; SAs/ex: severe asthma smokers/ex-smokers. | | | | |
