# Supplementary material for: Enhanced oxidative stress in smoking and ex-smoking severe asthma in the U-BIOPRED cohort
Source: PLoS One. 2018 Sep 21;13(9):e0203874. doi: 10.1371/journal.pone.0203874 (PMC6150501; doi:10.1371/journal.pone.0203874)
Supplement: S2 Table — (DOCX) [file pone.0203874.s002.docx]

**Table S2. Clinical and inflammatory characteristics of subjects present in the induced sputum-transcriptomics subset.**

|  | SAn | SAs/ex | *p*-value | |
| --- | --- | --- | --- | --- |
| Subjects *n*. | 61 | 23 | |  |
| Age (yr) | 55 (46-62) [*n*=61] | 54 (46-56) [*n*=23] | | 0.797 |
| Female | 37/61 (60.66%) | 12/23 (52.17%) | | 0.483 |
| Age at Diagnosis (yr) | 22 (6-38) [*n*= 60] | 39 (20-50) [*n*=23] | | **0.031** |
| Exacerbations (History) | 2 (1-3) [*n*=61] | 2 (0-3) [*n*=23] | | 0.575 |
| Pack Years | 1.50 (1-3) [*n*=14] | 16 (12-25) [*n*=23] | | **<0.001** |
| Allergic Rhinitis Diagnosed | 24/53 (45.28%) | 7/21 (33.33%) | | 0.350 |
| Nasal Polyps Diagnosed | 22/59 (37.29%) | 8/22 (36.36%) | | 0.939 |
| GERD Diagnosed | 25/58 (43.1%) | 13/21 (61.9%) | | 0.144 |
| FEV_1_ % pred | 61.25±2.77[*n*=61] | 67.74±3.61 [*n*=23] | | 0.129 |
| FVC % pred | 87.25±2.37[*n*=61] | 94.69±3.91 [*n*=23] | | 0.111 |
| FEV_1_/FVC ratio | 0.57±0.02 [*n*=61] | 0.59±0.02 [*n*=23] | | 0.560 |
| Exhaled NO | 24.50 (16-46) [*n*=59] | 28.50 (10-50) [*n*=22] | | 0.393 |
| Sputum Eosinophils | 14 (2-82) [*n*=61] | 36 (6-84) [*n*=23] | | 0.304 |
| Sputum Neutrophils | 312 (222-442) [*n*=61] | 296 (236-370) [*n*=23] | | 0.754 |
| Sputum Eosinophils (%) | 2.70 (0-16) [*n*=61] | 7.06 (1-16) [*n*=23] | | 0.384 |
| Sputum Neutrophils (%) | 66.12 (45-84) [*n*=61] | 55.15 (45-66) [*n*=23] | | 0.906 |
| Mean ACQ with ACQ7 | 2.43 (2-4) [*n*=57] | 2.21 (2-3) [*n*=20] | | 0.767 |
| Regular ICS or ICS/LABA Use | 61/61 (100%) | 23/23 (100%) | | 1.000 |
| Regular Oral Corticosteroids | 25/59 (42.37%) | 13/21 (61.90%) | | 0.128 |
| Data are presented as mean±SE [*n*], median (interquartile range) [*n*] or *n*/N (%), unless otherwise stated. ACQ: Asthma Control Questionnaire; FEV_1_: forced expiratory volume in 1 second; FVC: forced vital capacity; GERD: gastro-esophageal reflux disease; ICS: inhaled corticosteroids; LABA: long-acting β_2_-agonist; SAn: severe asthma non-smokers; SAs/ex: severe asthma smokers/ex-smokers. | | | | |
