# Supplementary material for: Enhanced oxidative stress in smoking and ex-smoking severe asthma in the U-BIOPRED cohort
Source: PLoS One. 2018 Sep 21;13(9):e0203874. doi: 10.1371/journal.pone.0203874 (PMC6150501; doi:10.1371/journal.pone.0203874)
Supplement: S3 Table — (DOCX) [file pone.0203874.s003.docx]

**Table S3**. **Clinical and inflammatory characteristics of subjects present in Bronchial Biopsy-transcriptomics subset.**

|  | SAn | SAs/ex | *p*-value | |
| --- | --- | --- | --- | --- |
| Subjects *n*. | 40 | 13 | |  |
| Age (yr) | 51 (40-60) [*n*=40] | 55 (52-56) [*n*=13] | | 0.152 |
| Female | 23/40 (57.5%) | 5/13 (38.46%) | | 0.237 |
| Age at Diagnosis (yr) | 9 (2-38) [*n*=39] | 33 (7-45) [*n*=13] | | 0.227 |
| Exacerbations (History) | 2 (1-4) [*n*=39] | 3 (1-4) [*n*=13] | | 0.362 |
| Pack Years | 1.75 (1-3) [*n*=6] | 22.50 (18-33) [*n*=13] | | **<0.001** |
| Allergic Rhinitis Diagnosed | 23/37 (62.16%) | 4/13 (30.77%) | | 0.058 |
| Nasal Polyps Diagnosed | 14/38 (36.84%) | 5/13 (38.46%) | | 0.917 |
| GERD Diagnosed | 21/39 (53.85%) | 8/12 (66.67%) | | 0.436 |
| FEV_1_ % pred | 72.19±3.25 [*n*=40] | 68.74±4.16 [*n*=13] | | 0.716 |
| FVC % pred | 90.04±2.81 [*n*=40] | 93.98±2.33 [*n*=13] | | 0.336 |
| FEV_1_/FVC ratio | 0.67±0.02 [*n*=40] | 0.60±0.03 [*n*=13] | | 0.074 |
| Exhaled NO | 30 (20-46) [*n*=35] | 19 (8-24) [*n*=13] | | **0.017** |
| Sputum Eosinophils | 14.50 (3-72) [*n*=22] | 6.50 (2-14) [*n*=4] | | 0.337 |
| Sputum Neutrophils | 267.50 (215-351)[*n*=22] | 260.00 (154-347) [*n*=4] | | 0.091 |
| Sputum Eosinophils (%) | 2.70 (1-14) [*n*=22] | 1.30 (0-3) [*n*=4] | | 0.321 |
| Sputum Neutrophils (%) | 51.47 (41-66) [*n*=22] | 49.23 (27-69) [*n*=4] | | 0.101 |
| Mean ACQ with ACQ7 | 2 (1-3) [*n*=34] | 2.79 (2-4) [*n*=10] | | 0.247 |
| Regular ICS or ICS/LABA Use | 40/40 (100%) | 13/13 (100%) | | 1.000 |
| Regular Oral Corticosteroids | 17/37 (45.95%) | 5/13 (38.46%) | | 0.641 |
| Data are presented as mean±SE [*n*], median (interquartile range) [*n*] or *n*/N (%), unless otherwise stated. ACQ: Asthma Control Questionnaire; FEV_1_: forced expiratory volume in 1 second; FVC: forced vital capacity; GERD: gastro-esophageal reflux disease; ICS: inhaled corticosteroids; LABA: long-acting β_2_-agonist; SAn: severe asthma non-smokers; SAs/ex: severe asthma smokers/ex-smokers. | | | | |
