# Supplementary material for: Enhanced oxidative stress in smoking and ex-smoking severe asthma in the U-BIOPRED cohort
Source: PLoS One. 2018 Sep 21;13(9):e0203874. doi: 10.1371/journal.pone.0203874 (PMC6150501; doi:10.1371/journal.pone.0203874)
Supplement: S4 Table — (DOCX) [file pone.0203874.s004.docx]

**Table S4**. **Clinical and inflammatory characteristics of subjects present in Bronchial Brushings-transcriptomics subset.**

|  | SAn | SAs/ex | *p*-value |
| --- | --- | --- | --- |
| Subjects *n*. | 49 | 18 |  |
| Age (yr) | 51 (38-60) [*n*=49] | 53 (48-55) [*n*=18] | 0.227 |
| Female | 25/49 (51.02%) | 7/18 (38.89%) | 0.380 |
| Age at Diagnosis (yr) | 15 (4-38) [*n*=47] | 31.5 (16-44) [*n*=18] | 0.165 |
| Exacerbations (History) | 2 (1-3) [*n*=48] | 2 (1-4) [*n*=18] | 0.804 |
| Pack Years | 2 (2-4) [*n*=9] | 20.25 (16-37) [*n*=18] | **<0.001** |
| Allergic Rhinitis Diagnosed | 27/44 (61.36%) | 6/18 (33.33%) | **0.049** |
| Nasal Polyps Diagnosed | 18/44 (40.91%) | 7/17 (41.18%) | 0.985 |
| GERD Diagnosed | 27/46 (58.7%) | 11/17 (64.71%) | 0.666 |
| FEV_1_ % pred | 75.78±2.4 [*n*=49] | 66.24±3.53 [*n*=18] | 0.134 |
| FVC % pred | 91.92±1.93 [*n*=49] | 90.11±2.61 [*n*=18] | 0.842 |
| FEV_1_/FVC ratio | 0.68±0.02 [*n*=49] | 0.60±0.02 [*n*=18] | **0.027** |
| Exhaled NO | 32.50 (19-56) [*n*=45] | 20.00 (10-28) [*n*=17] | **0.020** |
| Sputum Eosinophils | 7.00 (2-82) [*n*=21] | 3.00 (2-56) [*n*=7] | 0.783 |
| Sputum Neutrophils | 263 (214-420) [*n*=21] | 244 (193-329) [*n*=7] | 0.771 |
| Sputum Eosinophils (%) | 1.44 (0-16) [*n*=21] | 0.60 (0-10) [*n*=7] | 0.754 |
| Sputum Neutrophils (%) | 52.29 (40-71) [*n*=21] | 43.65 (33-64) [*n*=7] | 0.635 |
| Mean ACQ with ACQ7 | 2.00 (1-3) [*n*=39] | 2.57 (2-4) [*n*=13] | 0.330 |
| Regular ICS or ICS/LABA Use | 49/49 (100%) | 18/18 (100%) | 1.000 |
| Regular Oral Corticosteroids | 19/45 (42.22%) | 9/17 (52.94%) | 0.451 |
| Data are presented as mean±SE [*n*], median (interquartile range) [*n*] or *n*/N (%), unless otherwise stated. GERD: gastro-esophageal reflux disease; FEV_1_: forced expiratory volume in 1 second; FVC: forced vital capacity; ACQ: Asthma Control Questionnaire; ICS: inhaled corticosteroids; LABA: long-acting β_2_-agonist; SAn: severe asthma non-smokers; SAs/ex: severe asthma smokers/ex-smokers. | | | |
