# Supplementary material for: Enhanced oxidative stress in smoking and ex-smoking severe asthma in the U-BIOPRED cohort
Source: PLoS One. 2018 Sep 21;13(9):e0203874. doi: 10.1371/journal.pone.0203874 (PMC6150501; doi:10.1371/journal.pone.0203874)
Supplement: S5 Table — (DOCX) [file pone.0203874.s005.docx]

**S5 Table. U-BIOPRED Consortium Information**

**Lead author of the U-BIOPRED study group:**

Prof. Peter J. Sterk (MD, PhD)

e-mail: p.j.sterk@amc.uva.nl

**Definition for the U-BIOPRED Supplementary authors**

Clinical site research leads

Platform leads

Data cleaning team

Scientific Board and Management Board members

Core project management staff

**Definition for the contributors list**

Significant involvement in the clinical study

**Use of list:**

This list is to be used for all non-core clinical papers.

**Instructions**

Follow up clinical papers should re-use the baseline cohort description paper lists, in order to recognize the clinical staff involved in the study.

| **U-BIOPRED Supplementary authors** | |
| --- | --- |
| **Name** | **Affiliation** |
| Adcock I M | National Heart and Lung Institute, Imperial College, London, UK; |
| Auffray C | European Institute for Systems Biology and Medicine, CNRS-ENS-UCBL-INSERM, Lyon, France; |
| Bakke P | Department of Clinical Science, University of Bergen, Bergen, Norway; |
| Bansal A T | Acclarogen Ltd, St. John’s Innovation Centre, Cambridge, UK; |
| Baribaud F | Janssen R&D, USA; |
| Bates S | Respiratory Therapeutic Unit, GSK, London, UK; |
| Bel E H | Academic Medical Centre, University of Amsterdam, Amsterdam, The Netherlands; |
| Bigler J | *Previously Amgen Inc* |
| Bisgaard H | COPSAC, Copenhagen Prospective Studies on Asthma in Childhood, Herlev and Gentofte Hospital,  University of Copenhagen, Copenhagen, Denmark |
| Boedigheimer M J | Amgen Inc.; Thousand Oaks, USA |
| Bønnelykke K | COPSAC, Copenhagen Prospective Studies on Asthma in Childhood, Herlev and Gentofte  Hospital, University of Copenhagen, Copenhagen, Denmark; |
| Brandsma J | University of Southampton, Southampton, UK |
| Brinkman P | Academic Medical Centre, University of Amsterdam, Amsterdam, The Netherlands; |
| Bucchioni E | Chiesi Pharmaceuticals SPA, Parma, Italy |
| Burg D | Centre for Proteomic Research, Institute for Life Sciences, University of Southampton, Southampton, UK |
| Bush A | National Heart and Lung Institute, Imperial College, London, UK; Royal Brompton and Harefield NHS trust, UK |
| Caruso M | Dept. Clinical and Experimental Medicine, University of Catania, Catania, Italy; |
| Chanez P | Assistance publique des Hôpitaux de Marseille - Clinique des bronches, allergies et sommeil, Aix Marseille Université, Marseille, France |
| Chung F K | National Heart and Lung Institute, Imperial College, London, UK; |
| Compton C H | Respiratory Therapeutic Unit, GSK, London, UK |
| Corfield J | Areteva R&D, Nottingham, UK; |
| D’Amico A | University of Rome ‘Tor Vergata’, Rome Italy; |
| Dahlen S E | Centre for Allergy Research, Karolinska Institutet, Stockholm, Sweden |
| De Meulder B | European Institute for Systems Biology and Medicine, CNRS-ENS-UCBL-INSERM, Lyon, France; |
| Djukanovic R | NIHR Southampton Respiratory Biomedical Research Unit and Clinical and Experimental Sciences, Southampton, UK; |
| Erpenbeck V J | Translational Medicine, Respiratory Profiling, Novartis Institutes for Biomedical Research, Basel, Switzerland; |
| Erzen D | Boehringer Ingelheim Pharma GmbH & Co. KG; Biberach, Germany |
| Fichtner K | Boehringer Ingelheim Pharma GmbH & Co. KG; Biberach, Germany |
| Fitch N | BioSci Consulting, Maasmechelen, Belgium; |
| Fleming L J | National Heart and Lung Institute, Imperial College, London, UK; Royal Brompton and Harefield NHS trust, UK |
| Formaggio E | *Previously CROMSOURCE, Verona Italy* |
| Fowler S J | Centre for Respiratory Medicine and Allergy, Institute of Inflammation and Repair, University of Manchester and University Hospital of South Manchester, Manchester Academic Health Sciences Centre, Manchester, United Kingdom |
| Frey U | University Children’s Hospital, Basel, Switzerland; |
| Gahlemann M | Boehringer Ingelheim (Schweiz) GmbH,Basel, Switzerland; |
| Geiser T | Department of Respiratory Medicine, University Hospital Bern, Switzerland; |
| Guo Y | Data Science Institute, Imperial College, London, UK; |
| Hashimoto S | Academic Medical Centre, University of Amsterdam, Amsterdam, The Netherlands; |
| Haughney J | International Primary Care Respiratory Group, Aberdeen, Scotland; |
| Hedlin G | Dept. Women’s and Children’s Health & Centre for Allergy Research, Karolinska Institutet, Stockholm, Sweden; |
| Hekking P W | Academic Medical Centre, University of Amsterdam, Amsterdam, The Netherlands; |
| Higenbottam T | Allergy Therapeutics, West Sussex, UK; |
| Hohlfeld J M | Fraunhofer Institute for Toxicology and Experimental Medicine, Hannover, Germany |
| Holweg C | Respiratory and Allergy Diseases, Genentech, San Francisco, USA |
| Horváth I | Semmelweis University, Budapest, Hungary |
| Howarth P | NIHR Southampton Respiratory Biomedical Research Unit, Clinical and Experimental Sciences and Human Development and Health, Southampton, UK |
| James A J | Centre for Allergy Research, Karolinska Institutet, Stockholm, Sweden; |
| Knowles R | Arachos Pharma, Stevenge, UK; |
| Knox A J | Respiratory Research Unit, University of Nottingham, Nottingham, UK; |
| Krug N | Fraunhofer Institute for Toxicology and Experimental Medicine, Hannover, Germany; |
| Lefaudeux D | European Institute for Systems Biology and Medicine, CNRS-ENS-UCBL-INSERM, Lyon, France; |
| Loza M J | Janssen R&D, USA; |
| Lutter R | Academic Medical Centre, University of Amsterdam, Amsterdam, The Netherlands; |
| Masefield S | European Lung Foundation, Sheffield, UK; |
| Matthews J G | Respiratory and Allergy Diseases, Genentech, San Francisco, USA; |
| Mazein A | European Institute for Systems Biology and Medicine, CNRS-ENS-UCBL-INSERM, Lyon, France |
| Meiser A | Data Science Institute, Imperial College, London, UK |
| Middelveld R J M | Centre for Allergy Research, Karolinska Institutet, Stockholm, Sweden |
| Miralpeix M | Almirall, Barcelona, Spain; |
| Montuschi P | Università Cattolica del Sacro Cuore, Milan, Italy; |
| Mores N | Università Cattolica del Sacro Cuore, Milan, Italy; |
| Murray C S | Centre for Respiratory Medicine and Allergy, Institute of Inflammation and Repair, University of Manchester and University Hospital of South Manchester, Manchester Academic Health Sciences Centre, Manchester, United Kingdom |
| Musial J | Dept. of Medicine, Jagiellonian University Medical College, Krakow, Poland |
| Myles D | Respiratory Therapeutic Unit, GSK, London, UK; |
| Pahus L | Assistance publique des Hôpitaux de Marseille, Clinique des bronches, allergies et sommeil  Espace Éthique Méditerranéen, Aix-Marseille Université, Marseille, France; |
| Pavlidis S | National Heart and Lung Institute, Imperial College, London, UK |
| Powel P | European Lung Foundation, Sheffield, UK; |
| Praticò G | CROMSOURCE, Verona, Italy |
| Puig Valls M | CROMSOURCE, Barcelona, Spain |
| Rao N | Janssen R&D, USA; |
| Riley J | Respiratory Therapeutic Unit, GSK, London, UK; |
| Roberts A | Asthma UK, London, UK; |
| Roberts G | NIHR Southampton Respiratory Biomedical Research Unit, Clinical and Experimental Sciences and Human Development and Health, Southampton, UK; |
| Rowe A | Janssen R&D, UK; |
| Sandström T | Dept of Public Health and Clinical Medicine, Umeå University, Umeå, Sweden; |
| Seibold W | Boehringer Ingelheim Pharma GmbH, Biberach, Germany |
| Selby A | NIHR Southampton Respiratory Biomedical Research Unit, Clinical and Experimental Sciences and Human Development and Health, Southampton, UK; |
| Shaw D E | Respiratory Research Unit, University of Nottingham, UK; |
| Sigmund R | Boehringer Ingelheim Pharma GmbH & Co. KG; Biberach, Germany |
| Singer F | University Children’s Hospital, Zurich, Switzerland; |
| Skipp P J | Centre for Proteomic Research, Institute for Life Sciences, University of Southampton, Southampton, UK |
| Sousa A R | Respiratory Therapeutic Unit, GSK, London, UK; |
| Sterk P J | Academic Medical Centre, University of Amsterdam, Amsterdam, The Netherlands; |
| Sun K | Data Science Institute, Imperial College, London, UK |
| Thornton B | MSD, USA |
| van Aalderen W M | Academic Medical Centre, University of Amsterdam, Amsterdam, The Netherlands; |
| van Geest M | AstraZeneca, Mölndal, Sweden; |
| Vestbo J | Centre for Respiratory Medicine and Allergy, Institute of Inflammation and Repair, University of Manchester and University Hospital of South Manchester, Manchester Academic Health Sciences Centre, Manchester, United Kingdom |
| Vissing N H | COPSAC, Copenhagen Prospective Studies on Asthma in Childhood, Herlev and Gentofte Hospital,  University of Copenhagen, Copenhagen, Denmark; |
| Wagener A H | Academic Medical Center Amsterdam, Amsterdam, The Netherlands |
| Wagers S S | BioSci Consulting, Maasmechelen, Belgium |
| Weiszhart Z | Semmelweis University, Budapest, Hungary; |
| Wheelock C E | Centre for Allergy Research, Karolinska Institutet, Stockholm, Sweden; |
| Wilson S J | Histochemistry Research Unit, Faculty of Medicine, University of Southampton, Southampton, UK; |

**Contributors**

| Ahmed H, European Institute for Systems Biology and Medicine, CNRS-ENS-UCBL-INSERM, Lyon, France; |
| --- |
| Aliprantis Antonios, Merck Research Laboratories, Boston, USA; |
| Allen David, North West Severe Asthma Network, Pennine Acute Hospital NHS Trust, UK |
| Alving Kjell, Dept Women’s & Children’s Health, Uppsala University, Uppsala, Sweden |
| Badorrek P, Fraunhofer ITEM; Hannover, Germany |
| Balgoma David, Centre for Allergy Research, Karolinska Institutet, Stockholm, Sweden |
| Ballereau S, European institute for Systems Biology and Medicine, University of Lyon, France |
| Barber Clair, NIHR Southampton Respiratory Biomedical Research Unit and Clinical and Experimental Sciences, Southampton, UK; |
| Bautmans An, MSD, Brussels, Belgium |
| Behndig AF, Umeå University, Umea, Sweden |
| Beleta Jorge, Almirall S.A., Barcelona, Spain; |
| Berglind A, MSD, Brussels, Belgium |
| Berton A, AstraZeneca, Mölndal, Sweden |
| Bochenek Grazyna, II Department of Internal Medicine, Jagiellonian University Medical College, Krakow, Poland; |
| Braun Armin, Fraunhofer Institute for Toxicology and Experimental Medicine, Hannover, Germany; |
| Campagna D, Department of Clinical and Experimental Medicine, University of Catania, Catania, Italy; |
| *Carayannopoulos Leon,* *Previously at: MSD, USA;* |
| Carvalho da Purificação Rocha João Pedro, Royal Brompton and Harefield NHS Foundation Trust, London, UK; |
| Casaulta C, University Children’s Hospital of Bern, Switzerland |
| Chaiboonchoe A, European Institute for Systems Biology and Medicine, CNRS-ENS-UCBL-INSERM, Lyon, France; |
| Chaleckis Romanas, Centre of Allergy Research, Karolinska Institutet, Stockholm, Sweden |
| Dahlén B, Karolinska University Hospital & Centre for Allergy Research, Karolinska Institutet, Stockholm, Sweden |
| De Alba Jorge, Almirall S.A., Barcelona, Spain; |
| De Lepeleire Inge, MSD, Brussels, BE |
| Dekker Tamara, Academic Medical Centre, University of Amsterdam, Amsterdam, The Netherlands; |
| Delin Ingrid, Centre for Allergy Research, Karolinska Institutet, Stockholm, Sweden |
| Dennison P, NIHR Southampton Respiratory Biomedical Research Unit, Clinical and Experimental Sciences, NIHR-Wellcome Trust Clinical Research Facility, Faculty of Medicine, University of Southampton, Southampton, UK; |
| Dijkhuis Annemiek, Academic Medical Centre, University of Amsterdam, Amsterdam, The Netherlands; |
| Draper Aleksandra, BioSci Consulting, Maasmechelen, Belgium; |
| Dyson K, CROMSOURCE; Stirling, UK |
| Edwards Jessica, Asthma UK, London, UK; |
| El Hadjam L, European Institute for Systems Biology and Medicine, University of Lyon |
| Emma Rosalia, Department of Clinical and Experimental Medicine, University of Catania, Catania, Italy; |
| Ericsson Magnus, Karolinska University Hospital, Stockholm, Sweden |
| Faulenbach C, Fraunhofer ITEM; Hannover, Germany |
| Flood Breda, European Federation of Allergy and Airways Diseases Patient’s Associations, Brussels, Belgium |
| Galffy G, Semmelweis University, Budapest, Hungary; |
| Gallart Hector, Centre for Allergy Research, Karolinska Institutet, Stockholm, Sweden |
| Garissi D, Global Head Clinical Research Division, CROMSOURCE, Italy |
| Gent J, Royal Brompton and Harefield NHS Foundation Trust, London, UK; |
| Gerhardsson de Verdier M, AstraZeneca; Mölndal, Sweden; |
| Gibeon D, National Heart and Lung Institute, Imperial College, London, UK; |
| Gomez Cristina, Centre for Allergy Research, Karolinska Institutet, Stockholm, Sweden |
| Gove Kerry, NIHR Southampton Respiratory Biomedical Research Unit and Clinical and Experimental Sciences, Southampton, UK; |
| Gozzard Neil, UCB, Slough, UK; |
| Guillmant-Farry E, Royal Brompton Hospital, London, UK |
| Henriksson E, Karolinska University Hospital & Karolinska Institutet, Stockholm, Sweden |
| Hewitt Lorraine, NIHR Southampton Respiratory Biomedical Research Unit, Southampton, UK |
| Hoda U, Imperial College, London, UK |
| Hu Richard, Amgen Inc. Thousand Oaks, USA |
| Hu Sile, National Heart and Lung Institute, Imperial College, London, UK; |
| Hu X, Amgen Inc.; Thousand Oaks, USA |
| Jeyasingham E, UK Clinical Operations, GSK, Stockley Park, UK |
| Johnson K, Centre for respiratory medicine and allergy, Institute of Inflammation and repair, University Hospital of South Manchester, NHS Foundation Trust, Manchester, UK |
| Jullian N, European Institute for Systems Biology and Medicine, University of Lyon |
| Kamphuis Juliette, Longfonds, Amersfoort, The Netherlands; |
| Kennington Erika J., Asthma UK, London, UK; |
| Kerry Dyson, CromSource, Stirling, UK; |
| Kerry G, Centre for respiratory medicine and allergy, Institute of Inflammation and repair, University Hospital of South Manchester, NHS Foundation Trust, Manchester, UK |
| Klüglich M, Boehringer Ingelheim Pharma GmbH & Co. KG; Biberach, Germany |
| Knobel Hugo, Philips Research Laboratories, Eindhoven, The Netherlands; |
| Kolmert Johan, Centre for Allergy Research, Karolinska Institutet, Stockholm, Sweden |
| Konradsen J R, Dept. Women’s and Children’s Health & Centre for Allergy Research, Karolinska Institutet, Stockholm, Sweden |
| Kots Maxim, Chiesi Pharmaceuticals, SPA, Parma, Italy; |
| Krueger L, University Children's Hospital Bern, Switzerland |
| Kuo Scott, National Heart and Lung Institute, Imperial College, London, UK; |
| Kupczyk Maciej, Centre for Allergy Research, Karolinska Institutet, Stockholm, Sweden |
| Lambrecht Bart, University of Gent, Gent, Belgium; |
| Lantz A-S, Karolinska University Hospital & Centre for Allergy Research, Karolinska Institutet, Stockholm, Sweden |
| Larsson L X, AstraZeneca, Mölndal, Sweden |
| Latzin P, University Children’s Hospital of Bern, Bern, Switzerland |
| Lazarinis N, Karolinska University Hospital & Karolinska Institutet, Stockholm, Sweden |
| Lemonnier N, European Institute for Systems Biology and Medicine, CNRS-ENS-UCBL-INSERM, Lyon, France |
| Lone-Latif Saeeda, Academic Medical Centre, University of Amsterdam, Amsterdam, The Netherlands; |
| Lowe L A, Centre for respiratory medicine and allergy, Institute of Inflammation and repair, University Hospital of South Manchester, NHS Foundation Trust, Manchester, UK |
| Marouzet Lisa, NIHR Southampton Respiratory Biomedical Research Unit, Southampton, UK |
| Martin Jane, NIHR Southampton Respiratory Biomedical Research Unit, Southampton, UK |
| Mathon Caroline, Centre of Allergy Research, Karolinska Institutet, Stockholm, Sweden |
| McEvoy L, University Hospital, Department of Pulmonary Medicine, Bern, Switzerland |
| Meah Sally, National Heart and Lung Institute, Imperial College, London, UK; |
| Menzies-Gow A, Royal Brompton and Harefield NHS Foundation Trust, London, UK; |
| *Metcalf Leanne, Previously at: Asthma UK, London, UK;* |
| Mikus Maria, Science for Life Laboratory & The Royal Institute of Technology, Stockholm, Sweden; |
| Monk Philip, Synairgen Research Ltd, Southampton, UK; |
| Naz Shama, Centre for Allergy Research, Karolinska Institutet, Stockholm, Sweden |
| Nething K, Boehringer Ingelheim Pharma GmbH & Co. KG; Biberach, Germany |
| Nicholas Ben, University of Southampton, Southampton, UK |
| Nihlén U, *Previously AstraZeneca; Mölndal, Sweden;* |
| Nilsson Peter, Science for Life Laboratory & The Royal Institute of Technology, Stockholm, Sweden; |
| Niven R, North West Severe Asthma Network, University Hospital South Manchester, UK |
| Nordlund B, Dept. Women’s and Children’s Health & Centre for Allergy Research, Karolinska Institutet, Stockholm, Sweden |
| Nsubuga S, Royal Brompton Hospital, London, UK |
| Östling Jörgen, AstraZeneca, Mölndal, Sweden; |
| Pacino Antonio, Lega Italiano Anti Fumo, Catania, Italy; |
| Palkonen Susanna, European Federation of Allergy and Airways Diseases Patient’s Associations, Brussels, Belgium. |
| Pellet J, European Institute for Systems Biology and Medicine, CNRS-ENS-UCBL-INSERM, Lyon, France |
| Pennazza Giorgio, University of Rome ‘Tor Vergata’, Rome Italy; |
| Petrén Anne, Centre for Allergy Research, Karolinska Institutet, Stockholm, Sweden |
| Pink Sandy, NIHR Southampton Respiratory Biomedical Research Unit, Southampton, UK |
| Pison C, European Institute for Systems Biology and Medicine, CNRS-ENS-UCBL-INSERM, Lyon, France |
| Postle Anthony, University of Southampton, UK |
| *Rahman-Amin Malayka, Previously at: Asthma UK, London, UK;* |
| Ravanetti Lara, Academic Medical Centre, University of Amsterdam, Amsterdam, The Netherlands; |
| Ray Emma, NIHR Southampton Respiratory Biomedical Research Unit, Southampton, UK |
| Reinke Stacey, Centre for Allergy Research, Karolinska Institutet, Stockholm, Sweden |
| *Reynolds Leanne, Previously at: Asthma UK, London, UK;* |
| Riemann K, Boehringer Ingelheim Pharma GmbH & Co. KG; Biberach, Germany |
| Robberechts Martine, MSD, Brussels, Belgium |
| Rocha J P, Royal Brompton and Harefield NHS Foundation Trust |
| Rossios C, National Heart and Lung Institute, Imperial College, London, UK; |
| Russell Kirsty, National Heart and Lung Institute, Imperial College, London, UK; |
| Rutgers Michael, Longfonds, Amersfoort, The Netherlands; |
| Santini G, Università Cattolica del Sacro Cuore, Milan, Italy; |
| Santoninco Marco, University of Rome ‘Tor Vergata’, Rome Italy; |
| Saqi M, European Institute for Systems Biology and Medicine, CNRS-ENS-UCBL-INSERM, Lyon, France |
| Schoelch Corinna, Boehringer Ingelheim Pharma GmbH & Co. KG, Biberach, Germany |
| Schofield James P. R., Centre for Proteomic Research, Institute for Life Sciences, University of Southampton, Southampton, UK |
| Scott S, North West Severe Asthma Network, Countess of Chester Hospital, UK |
| Sehgal N, North West Severe Asthma Network; Pennine Acute Hospital NHS Trust |
| Sjödin Marcus, Centre for Allergy Research, Karolinska Institutet, Stockholm, Sweden |
| Smids Barbara, Academic Medical Centre, University of Amsterdam, Amsterdam, The Netherlands; |
| Smith Caroline, NIHR Southampton Respiratory Biomedical Research Unit, Southampton, UK |
| Smith Jessica, Asthma UK, London, UK; |
| Smith Katherine M., University of Nottingham, UK; |
| Söderman P, Dept. Women’s and Children’s Health, Karolinska Institutet, Stockholm, Sweden |
| Sogbesan A, Royal Brompton and Harefield NHS Foundation Trust, London, UK; |
| Spycher F, University Hospital Department of Pulmonary Medicine, Bern, Switzerland |
| Staykova Doroteya, University of Southampton, Southampton, UK |
| Stephan S, Centre for respiratory medicine and allergy, Institute of Inflammation and repair, University Hospital of South Manchester, NHS Foundation Trust, Manchester, UK |
| Stokholm J, University of Copenhagen and Danish Pediatric Asthma Centre Denmark |
| Strandberg K, Karolinska University Hospital & Karolinska Institutet, Stockholm, Sweden |
| Sunther M, Centre for respiratory medicine and allergy, Institute of Inflammation and repair, University Hospital of South Manchester, NHS Foundation Trust, Manchester, UK |
| Szentkereszty M, Semmelweis University, Budapest, Hungary; |
| Tamasi L, Semmelweis University, Budapest, Hungary; |
| Tariq K, NIHR Southampton Respiratory Biomedical Research Unit, Clinical and Experimental Sciences, NIHR-Wellcome Trust Clinical Research Facility, Faculty of Medicine, University of Southampton, Southampton, UK; |
| Thörngren John-Olof, Karolinska University Hospital, Stockholm, Sweden |
| Thorsen Jonathan, COPSAC, Copenhagen Prospective Studies on Asthma in Childhood, Herlev and Gentofte  Hospital, University of Copenhagen, Copenhagen, Denmark; |
| Valente S, Università Cattolica del Sacro Cuore, Milan, Italy; |
| van de Pol Marianne, Academic Medical Centre, University of Amsterdam, Amsterdam ,The Netherlands; |
| van Drunen C M, Academic Medical Centre, University of Amsterdam, Amsterdam, The Netherlands; |
| *Versnel Jenny, Previously at: Asthma UK, London, UK;* |
| Vink Anton, Philips Research Laboratories, Eindhoven, The Netherlands; |
| von Garnier C, University Hospital Bern, Switzerland; |
| Vyas A, North west Severe Asthma Network, Lancashire Teaching Hospitals NHS Trust, UK |
| Wald Frans, Boehringer Ingelheim Pharma GmbH & Co. KG, Biberach, Germany |
| Walker Samantha, Asthma UK, London, UK; |
| Ward Jonathan, Histochemistry Research Unit, Faculty of Medicine, University of Southampton, Southampton, UK; |
| Wetzel Kristiane, Boehringer Ingelheim Pharma GmbH, Biberach, Germany |
| Wiegman Coen, National Heart and Lung Institute, Imperial College, London, UK; |
| Williams Siân, International Primary Care Respiratory Group, Aberdeen, Scotland; |
| Yang Xian, Data Science Institute, Imperial College, London, UK |
| Yeyasingham Elizabeth, UK Clinical Operations, GSK, Stockley Park, UK; |
| Yu W, Amgen Inc.; Thousand Oaks, USA |
| Zetterquist W, Dept. Women’s and Children’s Health & Centre for Allergy Research, Karolinska Institutet, Stockholm, Sweden |
| Zolkipli Z, NIHR Southampton Respiratory Biomedical Research Unit, Clinical and Experimental Sciences and Human Development and Health, Southampton, UK; |
| Zwinderman A H, Academic Medical Centre, University of Amsterdam, The Netherlands; |

| **Partner organisations** | |
| --- | --- |
| Novartis Pharma AG | University of Southampton, Southampton, UK |
| Academic Medical Centre, University of Amsterdam, Amsterdam, The Netherlands | Imperial College London, London, UK |
| University of Catania, Catania, Italy | University of Rome ‘Tor Vergata’, Rome, Italy |
| Hvidore Hospital, Hvidore, Denmark | Jagiellonian Univ. Medi.College, Krakow, Poland |
| University Hospital, Inselspital, Bern, Switzerland | Semmelweis University, Budapest, Hungary |
| University of Manchester, Manchester, UK | Université d’Aix-Marseille, Marseille, France |
| Fraunhofer Institute, Hannover, Germany | University Hospital, Umea, Sweden |
| Ghent University, Ghent, Belgium | Ctr. Nat. Recherche Scientifique, Villejuif, France |
| Università Cattolica del Sacro Cuore, Rome, Italy | University Hospital, Copenhagen, Denmark |
| Karolinska Institutet, Stockholm, Sweden | Nottingham University Hospital, Nottingham, UK |
| University of Bergen, Bergen, Norway | Netherlands Asthma Foundation, Leusden, NL |
| European Lung Foundation, Sheffield, UK | Asthma UK, London, UK |
| European. Fed. of Allergy and Airways Diseases Patients’ Associations, Brussels, Belgium | Lega Italiano Anti Fumo, Catania, Italy |
| International Primary Care Respiratory Group, Aberdeen, Scotland | Philips Research Laboratories, Eindhoven, NL |
| Synairgen Research Ltd, Southampton, UK | Aerocrine AB, Stockholm, Sweden |
| BioSci Consulting, Maasmechelen, Belgium | Almirall |
| AstraZeneca | Boehringer Ingelheim |
| Chiesi | GlaxoSmithKline |
| Roche | UCB |
| Janssen Biologics BV | Amgen NV |
| Merck Sharp & Dome Corp |  |

| **MEMBERS OF THE ETHICS BOARD** | | | |
| --- | --- | --- | --- |
| **Name** | **Task** | **Affiliation** | **e-mail** |
| Jan-Bas Prins | Biomedical research | LUMC/the Netherlands | J.B.Prins@lumc.nl |
| Martina Gahlemann | Clinical care | BI/Germany | Martina.Gahlemann@boehringer-ingelheim.com |
| Luigi Visintin | Legal affairs | LIAF/Italy | visintin@inrete.it |
| Hazel Evans | Paediatric care | Southampton/UK | hazel.evans@uhs.nhs.uk |
| Martine Puhl | Patient representation (co chair) | NAF/ the Netherlands | martine@puhl.nl |
| Lina Buzermaniene | Patient representation | EFA/Lithuania | lina.buzermaniene@pavb.lt |
| Val Hudson | Patient representation | Asthma UK | hudsonval7@gmail.com |
| Laura Bond | Patient representation | Asthma UK | lvbond22@googlemail.com |
| Pim de Boer | Patient representation and pathobiology | IND | deboer.pim@hetnet.nl |
| Guy Widdershoven | Research ethics | VUMC/the Netherlands | g.widdershoven@vumc.nl |
| Ralf Sigmund | Research methodology and biostatistics | BI/Germany | ralf.sigmund@boehringer-ingelheim.com |

| **THE PATIENT INPUT PLATFORM** | |
| --- | --- |
| **Name** | **Country** |
| Amanda Roberts | UK |
| David Supple (chair) | UK |
| Dominique Hamerlijnck | The Netherlands |
| Jenny Negus | UK |
| Juliёtte Kamphuis | The Netherlands |
| Lehanne Sergison | UK |
| Luigi Visintin | Italy |
| Pim de Boer (co-chair) | The Netherlands |
| Susanne Onstein | The Netherlands |

| **MEMBERS OF THE SAFETY MONITORING BOARD** | |
| --- | --- |
| **Name** | **Task** |
| William MacNee | Clinical care |
| Renato Bernardini | Clinical pharmacology |
| Louis Bont | Paediatric care and infectious diseases |
| Per-Ake Wecksell | Patient representation |
| Pim de Boer | Patient representation and pathobiology (chair) |
| Martina Gahlemann | Patient safety advice and clinical care (co-chair) |
| Ralf Sigmund | Bio-informatician |
