# Supplementary material for: Enhanced oxidative stress in smoking and ex-smoking severe asthma in the U-BIOPRED cohort
Source: PLoS One. 2018 Sep 21;13(9):e0203874. doi: 10.1371/journal.pone.0203874 (PMC6150501; doi:10.1371/journal.pone.0203874)
Supplement: S1 File — (DOCX) [file pone.0203874.s006.docx]

**S1 Material and Methods**

**Enhanced oxidative stress in smoking and ex-smoking severe asthma in the U-BIOPRED cohort: Materials and Methods**

**Authors**

R. Emma^1^, A.T. Bansal^2^, J. Kolmert^3,4^, C.E. Wheelock^3^, S.E. Dahlen^4^, M.J. Loza^5^, B. De Meulder^6^, D. Lefaudeux^6^, C. Auffray^6^, B. Dahlen^7^, P.S. Bakke^8^, P. Chanez^9^, S.J. Fowler^10^, I. Horvath^11^, P. Montuschi^12^, N. Krug^13^, M. Sanak^14^, T. Sandstrom^15^, D.E. Shaw^16^, L.J. Fleming^17^, R. Djukanovic^18^, P.H. Howarth^18^, F. Singer^19,20^, A.R. Sousa^21^, P.J. Sterk^22^, J. Corfield^23,24^, I. Pandis^25^, K.F. Chung^17^, I.M. Adcock^17^, R. Lutter^22^, L. Fabbella^1^, M. Caruso^1*^, *U-BIOPRED Study Group.*

**Affiliations**

1 Department of Clinical and Experimental Medicine – University of Catania – Catania (Italy)

2 Acclarogen Ltd, St John's Innovation Centre, Cambridge, CB4 0WS, UK.

3 Division of Physiological Chemistry 2, Department of Medical Biochemistry and Biophysics, Karolinska Institutet, Stockholm, Sweden

4 Centre for Allergy Research, Institute of Environmental Medicine, Karolinska Institutet, Stockholm, Sweden

5 Janssen Research & Development, LLC , Springhouse, PA, United States

6 European Institute for Systems Biology and Medicine, CNRS-ENS-UCBL-INSERM, CIRI-UMR5308, Lyon, France

7 Karolinska University Hospital & Centre for Allergy Research, Karolinska Institutet, Stockholm, Sweden

8 Department of Clinical Science, University of Bergen, Bergen, Norway

9 Département des Maladies Respiratoires, CIC Nord , INSERM U1067 Aix Marseille Université Marseille, France

10 Centre for Respiratory Medicine and Allergy, The University of Manchester, Manchester Academic Health Science Centre, University Hospital of South Manchester NHS Foundation Trust, Manchester, UK; Airways Clinic, Lancashire Teaching Hospitals NHS Foundation Trust, Preston, UK

11 Department of Pulmonology, Semmelweis University, Budapest, Hungary

12 Faculty of Medicine, Catholic University of the Sacred Heart, Rome, Italy

13 Fraunhofer Institute for Toxicology and Experimental Medicine Hannover, Germany

14 Department of Medicine, Jagiellonian University Medical School, Krakow, Poland

15 Dept of Public Health and Clinical Medicine, Medicine, Umeå University, Umeå, Sweden

16 Respiratory Research Unit, University of Nottingham, Nottingham, UK

17 National Heart & Lung Institute, Imperial College, London, UK

18 NIHR Southampton Respiratory Biomedical Research Unit, Clinical and Experimental Sciences, University of Southampton Faculty of Medicine, Southampton UK

19 University Children's Hospital Bern, Bern, Switzerland

20 University Children's Hospital Zurich, Zurich, Switzerland

21 Respiratory Therapy Unit, GlaxoSmithKline, London, UK

22 Dept of Respiratory Medicine, Academic Medical Centre, University of Amsterdam, Amsterdam, The Netherlands

23 AstraZeneca R&D, Mölndal, Sweden

24 Areteva R&D, Nottingham, UK

25 Data Science Institute, South Kensington Campus, Imperial College London, London, UK

**Subjects**

Participants with severe asthma were recruited from the following 16 clinical centres in 11 European countries [1]:

- Academic Medical Centre, University of Amsterdam, Amsterdam, The Netherlands
- University of Southampton, Southampton, United Kingdom
- Imperial College London, London, United Kingdom
- Centre for Allergy Research, Karolinska Institutet, Stockholm, Sweden
- University of Catania, Catania, Italy
- Department of Medicine Jagiellonian University Medical College, Krakow, Poland
- Semmelweis University Department of Pulmonology, Budapest, Hungary
- Respiratory Research Group University of Manchester, Manchester, United Kingdom
- Université de la Méditerranee, Marseille, France
- Nottingham University Hospitals, Centre for Respiratory Research, Nottingham, United Kingdom
- Haukeland University Hospital, Bergen, Norway
- University Hospital, Inselspital, Bern, Switzerland
- Fraunhofer Institute of Toxicology and Experimental Medicine, Hannover, Germany
- Department of Respiratory Medicine and Allergy, University Hospital, Umea, Sweden
- Hvidore Hospital, Hvidore, Denmark
- Department of Pharmacology, Faculty of Medicine Università Cattolica del Sacro Cuore, Rome, Italy.

Severe asthma was defined according to the U-BIOBRED international consensus criteria [2]. Prior to enrolment, participants with severe asthma were required to have been under follow-up by a respiratory physician for at least six months, during which time assessments had been undertaken to optimize asthma control and assess medication adherence.

The study was approved by the ethics committee for each participating clinical institution, and adhered to the standards set by International Conference on Harmonisation and Good Clinical Practice. It is registered on ClinicalTrials.gov, (Identifier: NCT01976767). All participants gave signed informed consent.

**Protocol and sample collection**

All enrolled subjects underwent a baseline visit to assess current health status, atopy and pulmonary function. Spirometry, reversibility test (post-bronchodilator) and fraction of exhaled nitric oxide level (FeNO) at 50 mL/sec were performed. Spirometry were performed according to current ATS/ERS guidelines [3,4]. Allergic status was assessed by skin prick test and measurement of total and specific IgE to six common aeroallergens. Questionnaire was administered to assess asthma control (ACQ with ACQ7). Urine samples and induced sputum (IS) were collected during the baseline visit. Urine, collected into sterile container, was divided in labelled tubes and stored at -20 °C until use. Before the visit, subjects were encouraged to refrain from drinking extra fluids as well as diuretic beverages. IS was obtained from severe asthmatic subjects using an induction protocol by nebulization of saline solutions with increasing concentrations of sodium chloride (0,9%, 3%, 4,5% of NaCl). Before the induction, peak expiratory flow (PEF) was measured both before and after the administration of 400 µg of salbutamol. Induction was stopped if PEF falls by 20% from the baseline. Expectorated sputum was collected with a cough in Petri dishes and placed on ice. Sputum cell pellet was obtained to perform microarray analysis and differential eosinophil and neutrophil counts following a standardised operating procedure. Briefly, the mucoid portion was dissolved in a solution with 6,3 mM dithioeriythritol (DTE) in HEPES buffered saline, and filtered (100 µm filter – BD Bioscience Falcon 352360) to remove un-solubilized mucus. The cell pellet was obtained after centrifugation of the filtrate, and was subsequently re-suspended in PBS. One aliquot of the cell suspension was processed for differential sputum cell count by cytospin (Shandon Cytospin 2). The remaining cell suspension was centrifuged and the cell pellet was re-suspended in RNAlater and stored at -80 °C until microarray analysis. Some subjects underwent an optional bronchoscopy visit during which bronchial biopsy (BB) and bronchial brushing (BBr) were obtained.

**Microarray assessment**

RNA from RNAlater-preserved IS, BB, and BBr samples were extracted using Qiagen miRNeasy kit and amplified with Nugen ovation pico WTA kit (NuGen Technologies; San Carlos, CA). The cDNA was analysed using the Affymetrix HG-U133+PM microarray platform (Affymetrix, Santa Clara, CA). CEL files were normalized, assessed for quality control to exclude technical outliers (chip image analysis, Affymetrix GeneChip QC, RNA degradation analysis, distribution analysis, principal components analysis, and correlation analysis), and re-normalized using the robust multi-array (RMA) method.

For IS, RNA was extracted from 362 samples, of which 228 had sufficient sample for microarray analysis. 15 of these samples failed QC metrics, 19 additional samples were excluded (not considered enrolled in adult cohorts), and 71 samples disqualified for squamous>30% or no differential counts available, with 123 quality samples remaining for subsequent analyses (84 samples of severe asthma subjects; remainder were for non-severe asthmatics and healthy).

For BB, RNA was extracted from 116 samples and had sufficient sample for microarray analysis. Seven of these samples failed QC metrics, and 1 additional sample was excluded (not considered enrolled in adult cohorts), with 108 quality samples remaining for subsequent analyses (53 samples of severe asthma subjects; remainder were for non-severe asthmatics and healthy).

For BBr, RNA was extracted from 159 samples, of which 158 had sufficient sample for microarray analysis. Six of these samples failed QC metrics, 3 additional samples were excluded (2 duplicate samples and 1 not considered enrolled in adult cohorts), with 149 quality samples remaining for subsequent analyses (67 samples of severe asthma subjects; remainder were for non-severe asthmatics and healthy).

A log2-intensity threshold was established as the limit of reliable quantification (LOD) based on the 90th percentile signal of merged nonspecific probesets distribution in the array and by the inflection point of maximum variance with decreasing signal in a standard deviation vs. mean intensity plot across all probe sets. Probe sets with mean log2 intensity above this threshold in at least one of the 4 study cohorts were considered quantifiable and included in subsequent analyses.

Batch effects from RNA processing sets were observed for the sputum and BBr datasets, with the batch effect adjusted in the data matrices using linear modelling of batch (as random factor) and cohort. For the sputum dataset, 3 subjects had duplicate samples, of which the mean of the log2 intensities, after RNA processing set batch adjustment, were used in the final analysis dataset.

**References**

1. Shaw DE, Sousa AR, Fowler SJ, Fleming LJ, Roberts G, Corfield J, et al. Clinical and inflammatory characteristics of the European U-BIOPRED adult severe asthma cohort. Eur Respir J. 2015;

2. Bel EH, Sousa A, Fleming L, Bush A, Chung KF, Versnel J, et al. Diagnosis and definition of severe refractory asthma: an international consensus statement from the Innovative Medicine Initiative (IMI). Thorax. 2011;66: 910–7.

3. Reddel HK, Taylor DR, Bateman ED, Boulet L-P, Boushey HA, Busse WW, et al. An official American Thoracic Society/European Respiratory Society statement: asthma control and exacerbations: standardizing endpoints for clinical asthma trials and clinical practice. Am J Respir Crit Care Med. American Thoracic Society; 2009;180: 59–99.

4. Miller MR, Hankinson J, Brusasco V, Burgos F, Casaburi R, Coates A, et al. Standardisation of spirometry. Eur Respir J. 2005;26: 319–38.
